# Red Blood Cell-Mimic Nanocatalyst Triggering Radical Storm to Augment Cancer Immunotherapy

**DOI:** 10.1007/s40820-022-00801-z

**Published:** 2022-02-05

**Authors:** Jiong Li, Sijia Wang, Xinyi Lin, Yanbing Cao, Zhixiong Cai, Jing Wang, Zhenxi Zhang, Xiaolong Liu, Ming Wu, Cuiping Yao

**Affiliations:** 1 Key Laboratory of Biomedical Information Engineering of Ministry of Education, Institute of Biomedical Photonics and Sensing, School of Life Science and Technology, Xi’an Jiaotong University, Xi’an, 710049 People’s Republic of China; 2 The United Innovation of Mengchao Hepatobiliary Technology Key Laboratory of Fujian Province, Mengchao Hepatobiliary Hospital of Fujian Medical University, Fuzhou, 350025 People’s Republic of China; 3 Mengchao Med-X Center, Fuzhou University, Fuzhou, 350116 People’s Republic of China

**Keywords:** Red blood cell mimic, Metal–organic framework, Nanocatalyst, Radical storm, Cancer immunotherapy

## Abstract

Red blood cells (RBCs) have recently emerged as promosing candidates for cancer treatment in terms of relieving tumor hypoxia and inducing oxidative damage against cancer cells, but they are still far from satisfactory due to their limited oxygen transport and reactive oxygen species generation rate in tumor tissue. Herein, artificial RBCs (designated FTP@RBCM) with radical storm production ability were developed for oncotherapy through multidimensional reactivity pathways of Fe-protoporphyrin-based hybrid metal–organic frameworks (FTPs, as the core), including photodynamic/chemodynamic-like, catalase-like and glutathione peroxidase-like activities. Meanwhile, owing to the advantages of long circulation abilities of RBCs provided by their cell membranes (RBCMs), FTP with a surface coated with RBCMs (FTP@RBCM) could enormously accumulate at tumor site to achieve remarkably enhanced therapeutic efficiency. Intriguingly, this ROS-mediated dynamic therapy was demonstrated to induce acute local inflammation and high immunogenic cancer death, which evoked a systemic antitumor immune response when combined with the newly identified T cell immunoglobulin and mucin-containing molecule 3 (Tim-3) checkpoint blockade, leading to not only effective elimination of primary tumors but also an abscopal effect of growth suppression of distant tumors. Therefore, such RBC-mimic nanocatalysts with multidimensional catalytic capacities might provide a promising new insight into synergistic cancer treatment.

**Supplementary Information:**

The online version contains supplementary material available at 10.1007/s40820-022-00801-z.

## Highlights

- A red blood cell-mimic nanocatalyst with photodynamic/chemodynamic-like, catalase-like and glutathione peroxidase-like activities was developed to boost radical storms for tumor eradication.
- Combined with the Tim-3 immune checkpoint blockade, such radical therapy can systematically evoke a robust systemic antitumor immune response to eliminate residual cancer cells.

**Supplementary Information:**

The online version contains supplementary material available at 10.1007/s40820-022-00801-z.

## Graphical abstract

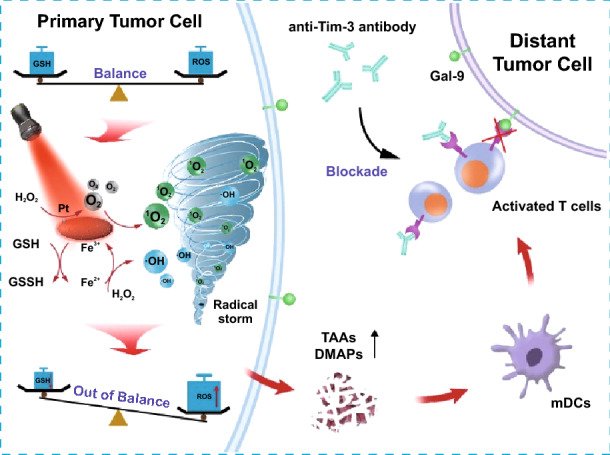

## Introduction

Red blood cells (RBCs), usually acting as oxygen transporters, can also carry out immunological surveillance through the generation of reactive oxygen species (ROS) from hemoglobin to remove certain bacterial pathogens in peripheral blood [1, 2]. The ROS generation ability of RBCs might be promising for other disease therapeutics, such as cancer radical therapy, but has not been exploited until now due to the limited ROS generation and the antioxidant defense system in cancer cells. Meanwhile, RBC-based delivery systems also encounter other limitations in cancer therapy: (1) their micrometer size hinders RBCs leaking from blood vessels and penetrating tumor tissues; (2) the risk of bacterial or virus contamination; and (3) other problems, including the need for rigorous storage conditions and harsh typing and cross-matching required to avoid mismatched transfusions [3]. Thus, the development of artificial RBC-based vehicles with oxygen delivery and radical storms that are free from the above shortcomings is highly desirable.

Radical therapies, mainly classified as photodynamic/sonodynamic/chemodynamic therapy (PDT, SDT, CDT) utilizing ROS to kill cancer cells, have been regarded as promising strategies for cancer treatment [4–6]. However, the efficacy of radical therapies is hampered by many factors, such as severe tumor hypoxia, shallow light penetration, rigorous Fenton reaction conditions (i.e., the requirement for enough H_2_O_2_ and strongly acidic conditions), a high level of intracellular reducing thiol species relevant to antioxidant defense, and so on [7]. It is thus extremely appealing to develop new strategies to overcome these drawbacks to promote the efficacy of radical therapies.

Recently, metal-organic framework (MOF) nanostructures have attracted intensive attention to augment ROS-relevant radical therapies. For instance, Fe-porphyrin-based-MOFs have emerged as highly effective photocatalysts for PDT with distinctive superiorities, such as high loading capacity without self-quenching, enhanced solubility and stability, and porous structures allowing the diffusion of ROS [8–10]. More importantly, the intracellular reducing thiol species can be depleted by Fe^3+^, and then the reduced Fe^2+^ can further conduct chemodynamic therapy (CDT) via an in situ Fenton or Fenton-like reaction that catalyzes overproduced H_2_O_2_ in the tumor microenvironment compared with normal tissues to generate hydroxyl radicals (·OH). All of these characteristics are favorable to aggravate oxidative damage to cancer cells [8]. Furthermore, the ingredients of Fe-porphyrin-based MOFs are nearly the same as protoheme (a key component of hemoglobin), which might ensure their endurable biocompatibility. Therefore, Fe-porphyrin-based MOFs have the potential to serve as red blood cell mimics to induce radical storms for tumor therapy.

In the process of eradicating cancer cells, radical-mediated dynamic therapies can effectively trigger immunogenic cell death (ICD) via the secretion of tumor-associated antigens (TAAs) and damage-associated molecular patterns (DAMPs), which then improve recruitment and antigen presentation to elicit antitumor immunity [11, 12]. Radical therapy has been demonstrated to potentiate the α-PD-1/L1 or CTLA-4 ICB, which not only suppresses locally treated tumors but also controls tumor growth at distant sites via abscopal effects. Unfortunately, α-PD-1/L1 or CTLA-4 therapies suffer from limited objective antitumor response rates and increased risks of intrinsic and adaptive resistance [13, 14]. Some solid tumors even remain refractory to either PD-1/L1 or CTLA-4 blockade, indicating an urgent need to explore the synergistic effect of radical therapy with other checkpoint inhibitors [15]. As a relatively newly identified immune checkpoint molecule, Tim-3 can be expressed by cytotoxic T cells, interferon-γ (IFN-γ)-producing helper T cells, dendritic cells (DCs), and many other lymphocyte subsets, thereby regulating immune responses by acting on multiple cell types [16, 17]. By binding to the main ligand of galectin-9, Tim-3 can directly lead to effector T cell exhaustion or apoptosis, or indirectly affect regulatory T cells and DCs to reduce CD8^+^ T cell cytotoxicity, thus impairing antitumor immune response [18–20]. Antibodies against Tim-3 blockade can significantly relieve tumor immunosuppression, and some of them have entered clinical trials [17, 21]. Thus, integrating Tim-3 blockade with radical therapy may significantly improve immunotherapy in multiple ways, including promoting antigen presentation, recovering T cell function, and reducing immunosuppressive cells to potentiate the antitumor immune response.

**Scheme 1:**
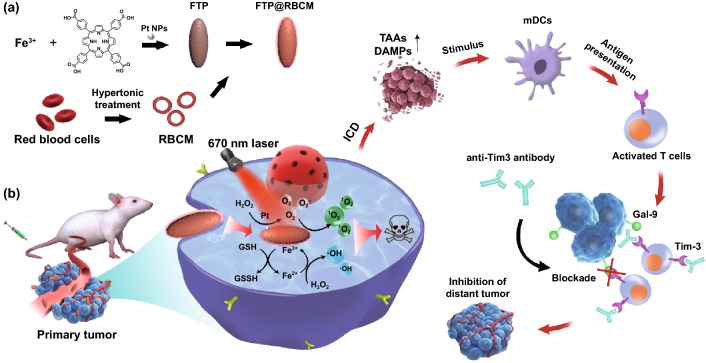
**a** Preparation of artificial RBCs (FTP@RBCM). **b** Immunotherapeutic mechanisms of the combination of radical therapy and Tim-3 checkpoint blockade

Based on the above context, we fabricated FTP@RBCM as artificial RBCs with a core of Fe-porphyrin-based MOFs doped with Pt nanoparticles and an outer layer of RBCMs, which can exert photodynamic/chemodynamic (PDT/CDT)-like, catalase-like and glutathione (GSH) peroxidase-like activities to augment Tim-3 blockade immunotherapy (Scheme 1). Compared with innate RBCs, the as-prepared artificial RBCs are free from the issues of large size, fragility, and feasibility while preserving long circulation abilities. Moreover, FTP@RBCM can strengthen O_2_ self-supply and ROS generation to boost radical storms to eliminate the primary tumor along with triggering acute local inflammation to induce ICD. Further combination of such radical therapy with Tim-3 blockade can systematically evoke intense antitumor immunity to suppress distant tumor growth. In general, this work presents a novel design of RBC-mimic nanocatalysts with various enzyme-like activities, which has huge potential for synergistic cancer immunotherapy.

## Experimental Section

See the Supporting Information for the experimental details.

## Results and Discussion

### Fabrication and Characterization of FTP@RBCM

**Fig. 1:**
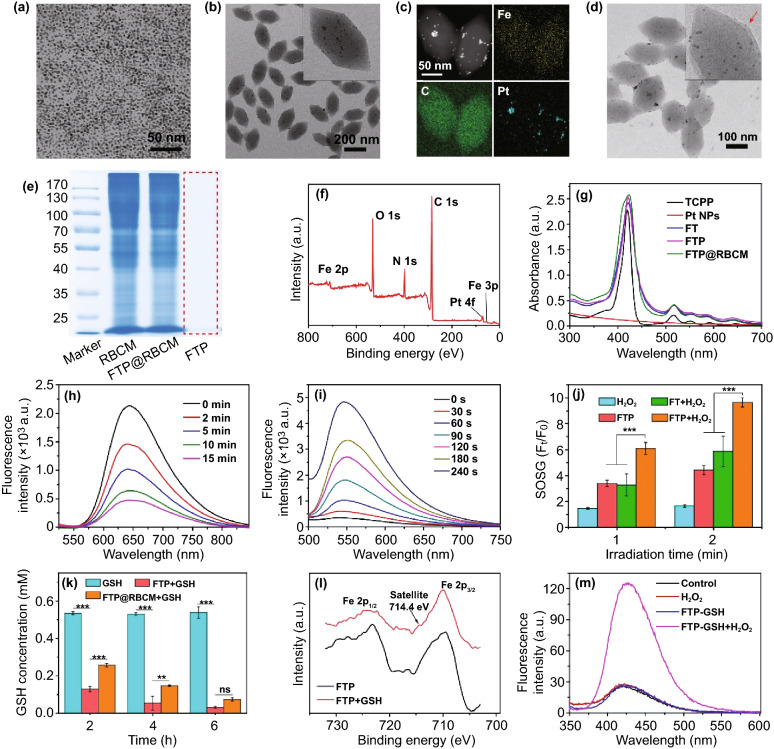
TEM image of **a** Pt nanoparticles and **b** FTP. **c** TEM mapping of FTP. **d** TEM image of FTP@RBCM. **e** SDS-PEGA protein analysis of RBCM, FTP, and FTP@RBCM. **f** XPS spectrum of FTP@RBCM. **g** UV–Vis spectra. **h** Fluorescence spectrum of [Ru(dpp)_3_]Cl_2_ incubated with FTP and H_2_O_2_. **i** Fluorescence spectrum of SOSG in the presence of FTP under 670 nm laser irradiation. **j** Relative fluorescence intensity of SOSG at 530 nm (*n* = 3). **k** GSH concentration after different treatments (*n* = 3). **l** XPS spectra of Fe 2p in FTP. **m** Fluorescence intensity of the solution containing TPA with different treatments

Pt nanoparticles (Pt NPs) were synthesized according to a previously reported method [22]. As shown in Fig. 1a, Pt nanoparticles had good dispersibility with an average diameter of approximately 3 nm. The non-Pt NP-doped MOFs (denoted FTs) were first prepared via a one-pot solvothermal method according to previous literature with some modifications [23]. For the synthesis of Pt NP-hybridized MOFs (denoted as FTPs), the aforementioned Pt NPs were mixed in advance with a reaction mixture to construct MOFs. As shown in Figs. S1 and 1b, the transmission electron microscopy (TEM) images of FTs and FTPs both exhibited uniform nanoshuttle morphology. The decoration of Pt NPs in FTPs could be clearly observed from the enlarged TEM image (the inset image in Fig. 1b), which was further confirmed by a TEM mapping assay (Fig. 1c). The dimensions of the FTPs were measured and calculated by Image J software to be approximately 220 ± 31 nm in length and 106 ± 15 nm in width. After red blood cell membrane camouflage, the biomimetic nanosystem still maintained excellent dispersion and clearly displayed a layer of RBCMs (Fig. 1d). The dimensions of FTP@RBCM increased to 223 ± 24 nm in length and 110 ± 19 nm in width. Additionally, dynamic light scattering (DLS) revealed that the hydrodynamic diameter of FTP@RBCM increased slightly, while its zeta potential decreased to the same level of RBCM vesicles (Fig. S2) comparing to that of the uncoated FTP nanoparticles (Fig. S3). To further verify the RBCM coating and the protein components, Coomassie blue staining was carried out. Nearly identical electrophoresis patterns between RBCM and FTP@RBCM lysate in Fig. 1e verified the successful coating of RBCM and the protein components with no obvious changes after modification. Moreover, the existence of external RBCM could improve the colloid stability of FTP under physiological conditions, without visible aggregation in PBS compared with the naked FTP without RBCM coating (Fig. S4). X-ray photoelectron spectroscopy (XPS) analysis identified the chemical elements in FTP@RBCM. As illustrated in Fig. 1f, the characteristic peaks of Fe 2p, Fe 3p, and Pt 4f binding energy in the spectrum indicated that Fe and Pt elements were involved in the formation of FTP@RBCM. UV–Vis spectra were also recorded to confirm the composition of FTP@RBCM (Fig. 1g). As expected, the characteristic peaks at 415, 516, 550, 590, and 645 nm ascribed to the B- and Q-band absorption of TCPP also appeared in the spectrum of FTP@RBCM.

### Characterization of Multidimensional Reactivities

The oxygen generation capacity of FTP via catalyzing H_2_O_2_ was evaluated using [Ru(dpp)_3_]Cl_2_ as an oxygen detection probe, with the fluorescence sensitively quenched in the presence of oxygen molecules. As shown in Fig. 1h, the fluorescence intensity of [Ru(dpp)_3_]Cl_2_ (*E*_*x*_ = 480 nm) rapidly declined with increasing co-incubation time of FTP and H_2_O_2_ from 0 to 15 min. By comparison, the fluorescence spectra of the PBS, H_2_O_2_, FTP, or FT + H_2_O_2_ groups all exhibited no significant changes (Fig. S5). These results indicated the catalase-like property of FTP doped with Pt NPs for oxygen generation. Next, we assessed the singlet oxygen (^1^O_2_) yield of FTP by using SOSG as the probe. As shown in Figs. 1i and S6a, with the extension of irradiation time, the fluorescence intensity of SOSG probe increased obviously when the FTP was exposed to a 670 nm laser, and the relative fluorescence intensity (F_t_/F_0_) of SOSG probe increased more than 12 times after 240 s irradiation, with the pattern showing a nearly linear dependence on the exposure time, implying good photostability for continuous ^1^O_2_ generation (Fig. S6b). In addition, FTP could accurately respond to OFF–ON laser irradiation to generate ^1^O_2_ and still have the capacity to repeatedly produce ^1^O_2_ with several cycles of irradiation (Fig. S7). It is noteworthy that FTP with H_2_O_2_ under laser irradiation could produce nearly 2 times higher ^1^O_2_ than that without H_2_O_2_ or FT without Pt nanoparticle doping (Figs. 1j and S8), suggesting that the enhanced ^1^O_2_ generation benefited from the O_2_ self-supply. In addition, FTP could also exhibit glutathione (GSH) peroxidase-like activity to scavenge GSH. As shown in Fig. 1k, the concentration of GSH in solution sharply decreased by more than 75% after 2 h of co-incubation with FTP, which is much important for avoiding the fast clearance of ROS by intracellular reduced species such as GSH. We also compared the catalase- and GSH peroxidase-like activities of FTP before and after coating with RBCM. As shown in Figs. S5 and 1 k, FTP with or without RBCM coating could exhibit both catalase- and GSH peroxidase-like activities, but it was less obvious for FTP@RBCM as the RBCM shielding reduced the reaction rate. Meanwhile, the Fe^3+^ in FTP would also be reduced to Fe^2+^ along with the oxidative consumption of GSH. As shown in Fig. 1la new satellite peak appeared at approximately 714.4 eV corresponding to Fe 2*p* in the XPS spectrum, providing clear evidence of Fe^2+^ generation [24], which was further verified by the formation of orange complexes after *o*-phenanthroline addition (Fig. S9). The produced Fe^2+^ could subsequently catalyze H_2_O_2_ to generate ·OH through the Fenton reaction, which could be monitored by the hydroxylation of terephthalate (TPA). When mixing the reaction product of FTP and GSH with H_2_O_2_ in the solution containing TPA, their fluorescence intensity exhibited a sharp increase (Fig. 1m), suggesting the production of massive amounts of ·OH in the mixture. In addition, the chromogenic reaction of tetramethylbenzidine (TMB, forming a distinctive blue color upon oxidation to monitor ROS generation) also demonstrated the time-dependent generation of ROS reflected by the increase in TMB absorption at 652 nm (Fig. S10). Collectively, these results demonstrated that FTP could perform multidimensional catalysis reactions of photodynamic/chemodynamic (PDT/CDT)-like, catalase-like, and glutathione (GSH) peroxidase-like reactivities to trigger radical storms.

### Cellular Internalization

**Fig. 2:**
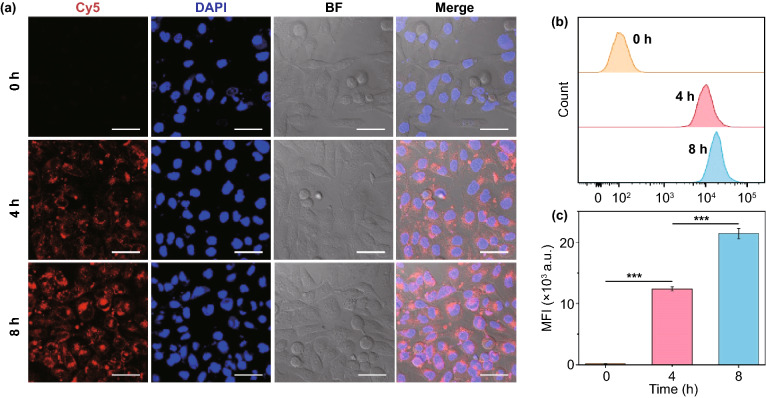
**a** CLSM images of Hep3B cells incubated with Cy5 labeled FTP and FTP@RBCM for 4 or 8 h. Scale bar = 50 µm. **b** Flow cytometry analysis of Cy5 fluorescence inside Hep3B cells. **c** The corresponding mean fluorescence intensity (MFI) of Cy5 in **b** (*n* = 3)

CD47 on the surface of red blood cells has been identified as a self-marker that displays a “Don’t eat me” signal to prevent macrophages from phagocytosing through binding with the signal regulatory protein-α (SIRP-α) receptor [25–28], as depicted in Fig. S11a. RBCM modification resulted in CD47 presentation on the surface of FTP@RBCM (Fig. S11b), thereby endowing it with the ability to escape from macrophages. As presented in Fig. S11c, the FTP group exhibited a strong red (Cy5) fluorescence intensity in RAW 264.7 murine macrophages; in contrast, the internalization efficiency of FTP@RBCM was dramatically decreased after RBCM coating. However, when the proteins on the RBCMs degenerated under high temperature at 80 °C for 10 min before being coated on the FTP, the phagocytosis of this degenerated membrane-coated FTP (defined as FTP@h-RBCM) significantly enhanced. These phenomena suggested the ability of FTP@RBCM to escape recognition and rapid elimination by the macrophages, which might extend their half-lives in blood and facilitate accumulation at the tumor site in vivo. We next evaluated the internalization of FTP@RBCM labeled by Cy5 in cancer cells. As shown in Fig. 2a, time-dependent uptake by Hep3B cells could be clearly observed by an increasing Cy5 fluorescence intensity, which demonstrated the efficient internalization of FTP@RBCM into cancer cells. Meanwhile, the curve of the flow cytometry analysis and quantification of the mean fluorescence intensity was consistent with the CLSM results (Fig. 2b, c).

### In Vitro Catalytic Activity and Killing Effect

**Fig. 3:**
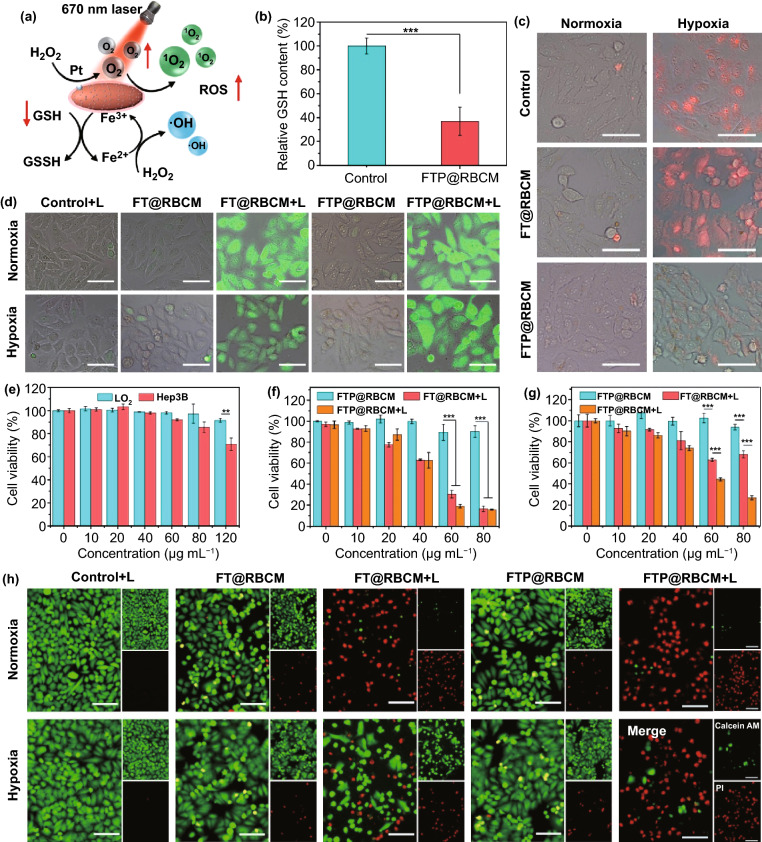
**a** Schematic illustration of the multiple catalytic activities of FTP@RBCM. **b** Relative GSH content of Hep3B cells with various treatments (*n* = 3). **c** Fluorescence microscopy images of probes [Ru(dpp)_3_]Cl_2_ to detect intracellular oxygen with various treatments. Scale bar = 50 µm. **d** Fluorescence microscopy images of DCFH-DA to detect intracellular ROS with various treatments. Scale bar = 50 µm. **e** Cell viabilities of LO2 and Hep3B cells after incubation with FTP@RBCM (*n* = 4). **f, g** Cell viabilities of Hep3B cells after different treatments under normoxia (**f**) or hypoxia (**g**) (*n* = 4). **h** Fluorescence microscope images of Hep3B cells stained by Calcein AM and PI. Scale bar = 100 µm

As a nanocatalyst with multidimensional activities, FTP@RBCM internalized by cancer cells could establish a series of catalytic reactions with H_2_O_2_ and GSH to increase the intracellular ROS level (Fig. 3a). First, we investigated the intracellular GSH depletion capacity of FTP@RBCM via a reduced GSH assay kit. As shown in Fig. 3b, the cellular GSH content dropped dramatically to approximately 36% after incubation with FTP@RBCM for 8 h, ascribed to the redox reaction between Fe^3+^ and GSH. Subsequently, intracellular hypoxic relief was explored by an oxygen detection probe [Ru(dpp)_3_]Cl_2_, with the fluorescence intensity positively correlated with the degree of intracellular hypoxia. As illustrated in Figs. 3c and S12, compared with the FT@RBCM counterpart without Pt NPs, which exhibited strong red fluorescence under hypoxic conditions, FTP@RBCM only showed slightly increased fluorescence when the culture conditions changed from normoxia to hypoxia, demonstrating that FTP@RBCM could relieve intracellular hypoxia to a large extent via catalase-like activity. Benefitting from GSH depletion and O_2_ self-supply, FTP@RBCM could induce enormous ROS generation and increase the sensitivity of cancer cells to oxidative damage under 670 nm laser exposure. Even under hypoxic conditions, the ROS detection probes in cells treated with FTP@RBCM and laser irradiation emitted comparably intense green fluorescence to that under normoxic conditions (Figs. 3d and S13), indicating a high ROS level in cells. Next, we further investigated the killing efficacy caused by FTP@RBCM in vitro by CCK-8 assay. The cancer cells (Hep3B) were more sensitive than normal cells (LO2) under FTP@RBCM treatment with no laser irradiation (Fig. 3e), as a high level of GSH in cancer cells promoted the reduction of Fe^3+^ to Fe^2+^, and concurrently the overproduced H_2_O_2_ offered the substrate of the Fenton reaction to generate more cytotoxic ·OH. Upon 670 nm laser irradiation, FT@RBCM and FTP@RBCM both had obvious cancer cell killing effects under normal O_2_ conditions, with cell viability declining below 80% at a TCPP concentration of 80 µg mL^−1^ (Fig. 3f). Intriguingly, FTP@RBCM still presented an outstanding PDT effect compared with FT@RBCM, which became incompetent under a hypoxic environment (Fig. 3g), due to the O_2_ self-supply capacity of FTP@RBCM. The result of the live/death cell staining assay (red color representing dead cells stained by PI, green color representing living cells stained by Calcein AM) was consistent with the CCK-8 assay (Fig. 3h), which also demonstrated that FTP@RBCM with versatile performances, including O_2_ self-supply, GSH depletion, CDT mediated by the Fenton reaction and PDT, could boost radical storms to kill cancer cells.

### Radical Therapy Induced Immunological Effect

**Fig. 4:**
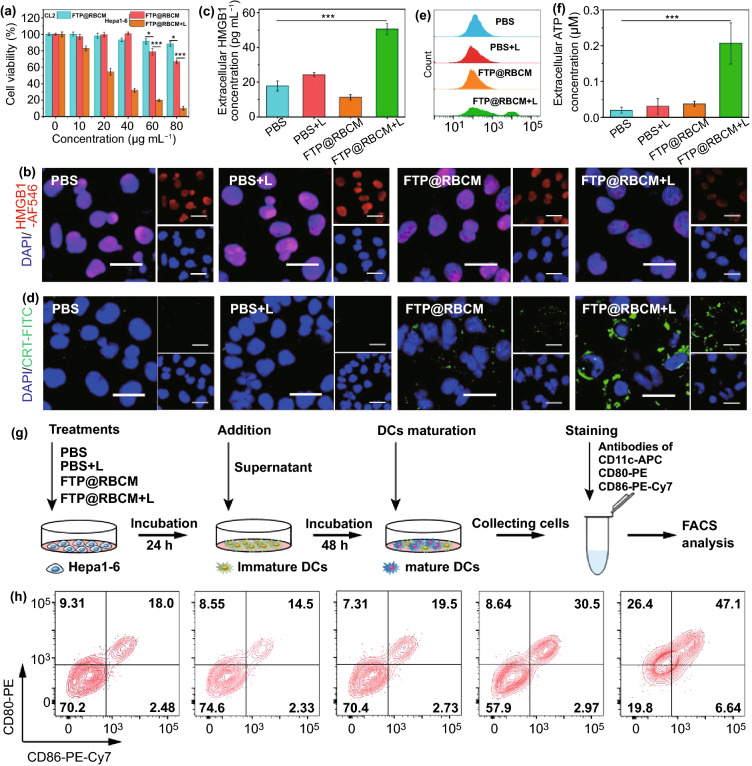
**a** Cell viabilities of CL2 and Hepa1-6 cells (*n* = 4). **b** CLSM images of HMGB1 immunofluorescence staining. Scale bar = 20 µm. **c** Extracellular HMGB1 levels (*n* = 3) **d** CLSM images of CRT immunofluorescence staining. Scale bar = 20 µm. **e** Flow cytometry analysis of CRT immunofluorescence staining. **f** Extracellular ATP levels (*n* = 3). **g** Schematic illustration of the experimental design and procedure of DC maturation assay in vitro. **h** DC maturation assay by flow cytometry

Radical therapy has been reported to induce ICD in cancer cells, and then evoke the body’s antitumor immune response [29, 30]. To explore the related mechanism, we selected the mouse hepatocellular carcinoma line Hepa1-6 to further investigate the immunological effect. First, we evaluated the killing effect of FTP@RBCM on Hepa1-6 cells using a CCK-8 assay. As shown in Fig. 4a, FTP@RBCM had relatively higher cytotoxicity toward Hepa1-6 cells than mouse normal CL2 cells, displaying a similar trend in the human hepatocellular carcinoma line Hep3B, as shown in Fig. 3e. After 670 nm laser irradiation, the cell viability of Hepa1-6 cells sharply decreased to below 10% at a TCPP concentration of 80 µg mL^−1^, verifying the good killing effect of FTP@RBCM on Hepa1-6 cells in vitro. Next, several ICD markers were studied via immunofluorescence staining kits. As shown in Figs. 4b and S14, the HMGB1 signals (indicated in red) in the cell nuclei drastically decreased in FTP@RBCM + L group compared with the other three groups, indicating that many HMGB1 molecules were released into the medium, which was further verified by extracellular HMGB1 detection (Fig. 4c). Meanwhile, strong CRT signals (indicated in green) were expressed on the cell membrane of Hepa1-6 cells treated with FTP@RBCM and laser irradiation, as evidenced by CLSM imaging (Figs. 4d and S15) and flow cytometry measurements (Figs. 4e and S16). In addition, ATP release is also an important characteristic of ICD, which can recruit immune cells and induce proinflammatory effects. Thus, we further detected the extra- and intracellular ATP levels of Hepa1-6 cells to evaluate the release of ATP after different treatments. As depicted in Figs. 4f and S17, the cells in FTP@RBCM + L group showed the most significant ATP secretion among all groups. The above results collectively suggested that FTP@RBCM under laser irradiation could effectively trigger radical storms and induce ICD in cancer cells to release DAMPs. Subsequently, we further investigated whether this therapeutic paradigm elicited antitumor immunity through the following experiment to check DC maturation. As illustrated in Fig. 4g, the supernatants of different treatment groups were added to immature DCs to stimulate their maturation for 48 h. As anticipated, the supernatant of Hepa1-6 cells in FTP@RBCM + L group could enormously facilitate DC maturation, with the highest percentage of CD80/CD86 expression among all experimental groups (Figs. 4h and S18).

### In Vivo Bio-Distribution and Radical Therapy

**Fig. 5:**
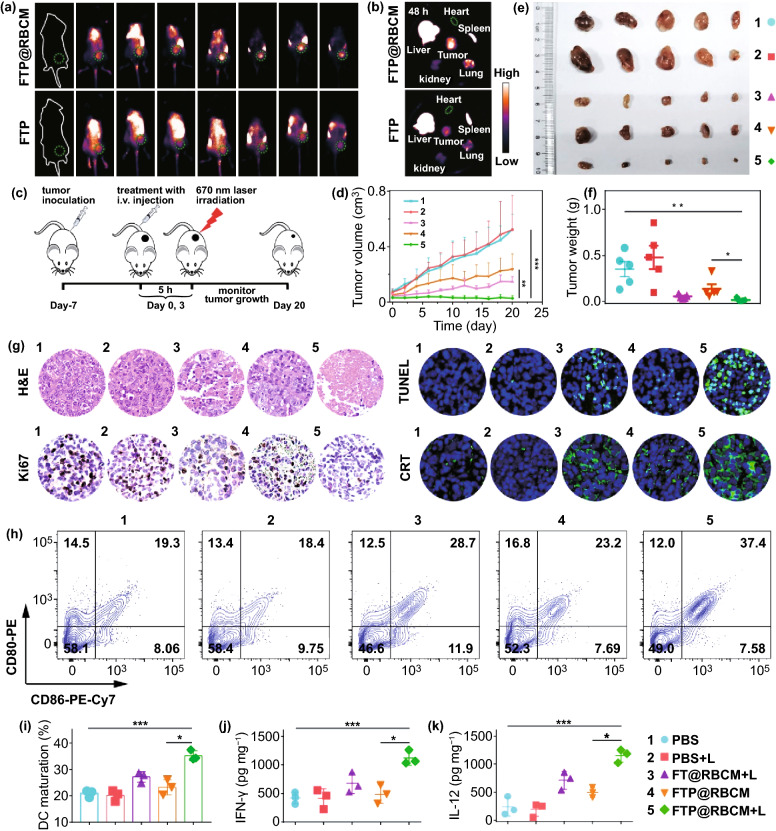
**a** Fluorescence images of mice injected with ICG labeled FTP or FTP@RBCM. **b** Fluorescence images of major organs and tumors. **c** Schematic illustration of the therapeutic procedure of FTP@RBCM. **d-f** Tumor growth curves (**d**), Ex vivo tumor photos (**e**) and average tumor weights (**f**) of mice (*n* = 5). **g** Optical microscope images of H&E and Ki67, staining, and TUNEL and CRT immunofluorescence staining. **h** DC maturation in draining inguinal lymph nodes. **i** Percentages of maturated DCs (CD11c^+^CD86^+^CD80^+^) (*n* = 3). **j, k** Cytokine levels of IFN-γ (**j**) and IL-12 (**k**) in tumor tissues (*n* = 3). 1: PBS group; 2: PBS + L group; 3: FT@RBCM + L group; 4: FTP@RBCM group; 5: FTP@RBCM + L group

Before evaluating the in vivo antitumor efficiency and immune response, we first investigated the blood compatibility of FTP@RBCM through a hemolysis test. As depicted in Fig. S19, the red blood cells showed no obvious hemolysis, and the hemolysis ratio calculated from the absorbance at 570 nm was less than 3% when the TCPP concentration in FTP@RBCM was up to 80 µg mL^−1^, demonstrating that FTP@RBCM with good blood compatibility was capable of intravenous administration. Subsequently, FTP@RBCM was labeled with indocyanine green (indicated as FTP@RBCM-ICG) to investigate the in vivo bio-distribution. As illustrated in Figs. 5a and S20, the indocyanine green (ICG) fluorescence signal at tumor site of the mouse continuously increased over time and reached a peak value at 5 h after *i.v.* injection. Notably, the ICG fluorescence signal at tumor site with *i.v.* injection of FTP@RBCM-ICG was significantly higher than that of FTP-ICG, which was attributed to the prolonged circulation conferred by RBCMs (Fig. S21). The excised major organs and tumor after 48 h of injection also demonstrated that FTP@RBCM-ICG could better accumulate at the tumor site with a more than 1.8 times stronger signal intensity than FTP-ICG (Figs. 5b and S22), which was beneficial for improving radical therapy in vivo.

Therefore, we assessed the antitumor effect induced by FTP@RBCM in Hepa1-6 tumor-bearing mice using the experimental procedures displayed in Fig. 5c. The 670 nm laser irradiation (100 mW cm^−2^, 10 min) was carried out at the time point of maximum accumulation of FTP@RBCM (Fig. S20). Then, tumor growth was monitored every other day for 20 days. As shown in Fig. 5d, the tumor volume of mice in FTP@RBCM + L group was obviously decreased compared with that of the other groups, which was attributed to the radical storms induced by FTP@RBCM with various catalytic activities in the TME. These results were further verified by excised tumor images and weights at the end of treatment (Fig. 5e–f). In addition, the tumor tissues after treatment were sectioned for H&E, Ki67, and TUNEL staining, demonstrating that the FTP@RBCM + L group showed severe histological damage, much less proliferation and a high degree of apoptosis, all being responsible for the tumor growth suppression (Figs. 5g and S23). In addition, there were no significant changes in the mouse weights among different groups during treatment, suggesting the low systematic toxicity of our artificial RBCs (Fig. S24). The safety of FTP@RBCM was further validated by H&E observation of major organs at the end of treatment with no obvious tissue damage (Fig. S25). Nevertheless, the significant accumulation of FTP@RBCM in the liver and spleen, as depicted in Fig. 5b, reminded us to pay more attention to long-term organ and system toxicity in future applications.

Furthermore, the CRT expression in the tumor tissues was analyzed by immunofluorescence staining to investigate whether radical therapy with FTP@RBCM could induce ICD in vivo. As anticipated, the tumor tissue of mice after receiving FTP@RBCM treatment under laser exposure showed a strong green fluorescence signal of CRT molecules (Figs. 5g and S23). Subsequently, we also evaluated DC maturation (CD11c^+^CD86^+^CD80^+^) in the draining inguinal lymph nodes of the mice with different treatments by flow cytometry. It is worth noting that the average percentage of DC maturation dramatically increased from approximately 20.2% in the PBS group to 34.8% in the FTP@RBCM + L group (Figs. 5h-i and S26), which indicated that the radical storms induced by nanocatalysts could effectively initiate antitumor immune responses, with IFN-γ and IL-12 levels in the tumor tissues significantly elevated by more than 2.6- and sixfold, respectively, compared with the PBS-treated group (Fig. 5j-k).

### In Vivo Synergetic Effect of Radical Therapy and Tim-3 ICB

**Fig. 6:**
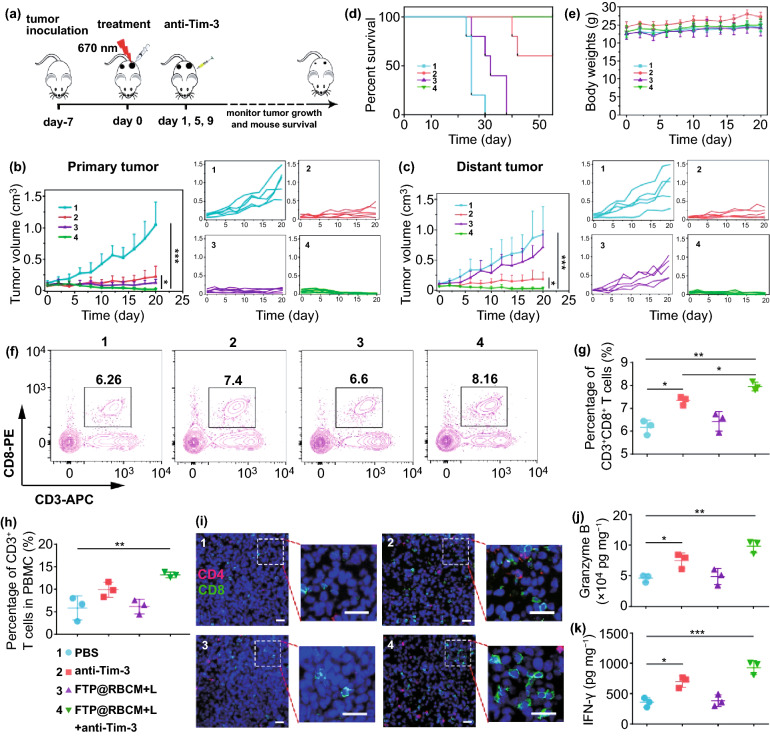
**a** Schematic illustration of combined therapeutic procedures of FTP@RBCM-mediated radical therapy combined with Tim-3 checkpoint blockade. **b, c** Relative growth and average relative growth curves of primary tumors (**b**) and abscopal tumors (**c**) (*n* = 5). **d** Survival curves of mice (*n* = 5). **e** Average body weights of mice (*n* = 5). **f** Representative flow cytometry plots of CD3^+^CD8^+^ T cells in spleen. **g** Statistical analysis of CD3^+^CD8^+^ T cell frequency according to the data in **f**. **h** Statistical analysis of CD3^+^ T cell frequency in PBMC. **i** Immunofluorescence staining images of the infiltrated CD4^+^ T cells (red) and CD8^+^ T cells (green) in abscopal tumor slices. Scale bar = 50 µm. **j, k** Cytokine levels of Granzyme B (**j**) and IFN-γ (**k**) in abscopal tumor tissues. 1: PBS group; 2: anti-Tim-3 group; 3: FTP@RBCM + L group; 4: FTP@RBCM + L + anti-Tim-3 group

Numerous studies have identified that the immune responses induced by radical therapy alone usually not strong enough to effectively eliminate residual tumors and inhibit distant tumors [31, 32]. To overcome this obstacle, we further combined radical therapy with the anti-Tim-3 antibody to systematically evoke robust antitumor immunity. For this purpose, we established a bilateral Hepa1-6 tumor model with primary and distant tumors. The detailed experimental procedures are depicted in Fig. 6a. Subsequently, the growth of the primary and abscopal tumors was monitored every other day. As shown in Fig. 6b–c, the primary tumors treated with anti-Tim-3 antibodies or radical therapy exhibited obvious tumor inhibition compared with the PBS group. Notably, the synergistic treatment group displayed the best antitumor efficiency among all groups. For abscopal tumor growth, the FTP@RBCM + L group showed negligible tumor inhibition in comparison with the PBS group, indicating that only radical therapy could not effectively inhibit distant tumor growth. However, abscopal tumors were almost completely suppressed when radical therapy was combined with Tim-3 blockade, which might be ascribed to the elevated systematic antitumor immunity. The survival of mice also indicated an excellent synergistic treatment effect, with 100% mouse survival after treatment for 55 days (Fig. 6d). Additionally, there was no significant weight loss of the mice in different groups, revealing few side effects of the treatments (Fig. 6e). Meanwhile, the serum biochemical indices in the experimental groups were also detected and found to show inappreciable changes compared with the PBS group, indicating no detectable hepatic and renal dysfunction of mice with various therapeutic schedules (Fig. S27).

To further evaluate the systematic immune responses and mechanisms underlying the prominent antitumor efficiency against tumor growth, the frequency of various lymphocytes in the tumors and spleens of mice with different treatments was analyzed. The results of lymphocyte analysis of the primary tumors showed that synergistic treatment could significantly improve the infiltration of CD8^+^ and CD4^+^ T cells (Fig. S28), repolarize macrophages from a pro-tumorigenic M2 phenotype to an antitumor M1 phenotype (Fig. S29), deplete myeloid-derived suppressor cells (MDSCs) (Fig. S30) and increase DC infiltration (Fig. S31). These results demonstrated that synergistic treatment could not only directly kill primary tumor cells via radical storms, but also simultaneously improve the intratumoral immune responses. Additionally, CD8^+^ T cells in spleens were also detected and displayed in Figs. 6f–g and S32, showing the synergistic treatment group had the highest ratio of CD8^+^ T cells in all groups. Meanwhile, synergistic treatment resulted in a significant shift of naive CD8^+^ T cells towards effector memory phenotypes (Fig. S33), which implied the activation of systemic immune response under a synergistic strategy, along with long-term immune memory formation. In addition, synergistic treatment with radical therapy and Tim-3 blockade also exhibited the highest percentage of CD3^+^ T cells in peripheral blood mononuclear cells (PBMCs) (Fig. 6h), which further verified the activation of systemic immunity.

The tumor-infiltrating lymphocytes of abscopal tumors were assessed via immunofluorescence staining (Figs. 6i and S34). As expected, the distant tumor tissue of the synergistic treatment group displayed more green signals representing CD8^+^ T cells than the other groups, with the number of infiltrated CD8^+^ T cells in group 4 (FTP@RBCM + L + anti-Tim-3 group) is about 2.5-folds higher than group 1 (PBS group), 1.5-folds higher than group 2 (anti-Tim-3 group), and 2-folds higher than group 3 (FTP@RBCM + L group). These results demonstrated the tremendous improvement of CD8^+^ T cell infiltration to distant tumors. Moreover, the secretion of immune-related cytokines in abscopal tumors also proved that synergistic therapy could boost immune responses against distant tumors, with the highest levels of proinflammatory factors of Granzyme B and IFN-γ among all groups (Fig. 6j–k). These results verified that radical storms triggered by artificial RBCs could strengthen Tim-3 ICB therapy, evoking systemic immunity to suppress distant tumors.

## Conclusions

In summary, we successfully prepared a red blood cell-mimic nanocatalyst constructed from Fe-porphyrin based hybrid MOFs and RBCMs. FTP@RBCM with various catalytic activities, including photodynamic/chemodynamic (PDT/CDT)-like, catalase-like and glutathione (GSH) peroxidase-like activities, can boost radical storms for primary tumor eradication. Meanwhile, the RBCM cloak provided the MOFs with enhanced colloidal stability, extended blood circulation, and improved tumor accumulation. Moreover, the severe ICD of cancer cells induced by our artificial RBCs can elicit systemic immune responses by releasing DAMPs and TAAs, which are further strengthened to effectively inhibit abscopal tumors in combination with Tim-3 blockade via triggering DC maturation, enhancing proinflammatory factor secretion, and promoting CD8^+^ T cell activation and infiltration. The synergistic radical immunotherapy effects and related mechanisms have been well characterized both in vitro and in vivo studies. Thus, the as-presented therapeutic paradigm of artificial RBCs for triggering radical storms to augment Tim-3 ICB is expected to provide a promising strategy for oncotherapy in future.

## Supplementary Information

Below is the link to the electronic supplementary material.

Supplementary file1 (PDF 2517 kb)
