# Supplementary material for: Red Blood Cell-Mimic Nanocatalyst Triggering Radical Storm to Augment Cancer Immunotherapy
Source: Nanomicro Lett. 2022 Feb 5;14:57. doi: 10.1007/s40820-022-00801-z (PMC8817004; doi:10.1007/s40820-022-00801-z)
Supplement: Supplementary file 1 — Supplementary file1 (PDF 2517 kb) [file 40820_2022_801_MOESM1_ESM.pdf]

Supporting Information for

Red Blood Cell-Mimic Nanocatalyst Triggering Radical Storm to Augment Cancer Immunotherapy

Jiong Li[1, #], Sijia Wang[1, #], Xinyi Lin[1], Yanbing Cao[2, 3], Zhixiong Cai[2, 3], Jing Wang[1], Zhenxi Zhang[1], Xiaolong Liu[2, 3], Ming Wu[2, 3, *], Cuiping Yao[1, *]

[1]Key Laboratory of Biomedical Information Engineering of Ministry of Education, Institute of Biomedical Photonics and Sensing, School of Life Science and Technology, Xi'an Jiaotong University, Xi'an 710049, P. R. China

[2]The United Innovation of Mengchao Hepatobiliary Technology Key Laboratory of Fujian Province, Mengchao Hepatobiliary Hospital of Fujian Medical University, Fuzhou 350025, P. R. China

[3]Mengchao Med-X Center, Fuzhou University, Fuzhou 350116, P. R. China

[#]Jiong Li and Sijia Wang contributed equally to this work

*Corresponding authors. E-mail: wmmj0419@163.com (Ming Wu), zsycp@xjtu.edu.cn (Cuiping Yao)

S1 Supplemental Text

S1.1 Materials

Iron chloride hexahydrate ($FeCl_3·6H_2O$), Tetrakis(4-carboxyphenyl) porphyrin (TCPP), benzoic acid, Chloroplatinic acid (($H_2PtCl_6·6H_2O$), Poly(vinylpyrrolidone) (PVP, MW = 55,000), 4,6-diamidino-2-phenylindole (DAPI), Tris (4, 7-diphenyl-1, 10-phenanthroline) ruthenium (II) dichloride complex ($[Ru(dpp)_3]Cl_2$) were obtained from Aladdin (Shanghai, PRC). 2,7-dichlorodihydrofluorescein diacetate (DCFH-DA), Calcein-AM and propidium iodide (PI) were purchased from J&K Scientific. Reduced Glutathione (GSH) Assay Kit was obtained from Solarbio Life Sciences (Beijing, China). Cell Counting Kit-8 (CCK-8) and Annexin V-FITC apoptosis detection kits were obtained from Dojindo Molecular Technologies (Tokyo, Japan). Deionized water (18.2 M Ω cm) was obtained using a Milli-Q Gradient System (Millipore Corporation, Bedford, MA, USA) and used for all the experiments. If not specified, all other chemicals were commercially available and used as received.

S1.2 Instruments

The morphologies of nanoparticles were observed using transmission electron microscopy (TEM, FEI Company, Hillsboro, OR). The UV-vis absorbance and fluorescence spectrum were measured by a Spectra Max M5 microplate reader (Molecular Devices, USA) and a Cary Eclipse fluorescence spectrophotometer (Agilent Technologies, Malaysia), respectively. The 670 nm laser (Diode Laser System, BWT Beijng Ltd.) was used to induce photodynamic therapy (PDT). Coomassie blue staining was observed under a ChemiDoc™ MP imaging system. The images of cell uptake were obtained by a CLSM (Zeiss LSM780). Live and death assay and ROS generation assay were observed by a fluorescence microscopy (Zeiss Axio Vert.A1, Germany). The mice imaging was performed on the UniNano-NIR II system. The frequencies of DC maturation and T cell infiltration were measured by a flow cytometry (BD FACSVerse™).

S1.3 Cell Culture

The human hepatocellular carcinoma line Hep3B cells, human normal liver cell line LO2 cells, murine hepatocellular carcinoma line Hepa1-6 cells and murine normal liver cell line CL2 cells were obtained from ATCC (Manassas, VA) and cultured in DMEM medium supplemented with 10% fetal bovine serum (FBS) at the humidified incubator with 5% CO_2.

S1.4 Synthesis of Pt Nanoparticles

The Pt nanoparticles were synthesized according to the previous literature. Briefly, 50.75 mg of $H_2PtCl_6{\cdot}6H_2O$ and 222 mg of poly(vinylpyrrolidone) (PVP) were added into 20 mL of ethylene glycol. The above homogeneous solution was heated to 180 °C and kept at this temperature for 20 minutes, then cooled down to room temperature. Subsequently, the as-synthesized PVP-protected Pt nanoparticles were precipitated in acetone and centrifugated at 10,000 rpm for 10 minutes. After being washed with acetone and hexane three times to remove excess free PVP, the Pt nanoparticles were re-dispersed in ethanol for further usage.

S1.5 Synthesis of FTP

30 mg of $FeCl_3{\cdot}6H_2O$ dissolved in 10 mL DMF and then added with 2 mg of Pt nanoparticles, 10 mg of TCPP and 280 mg of benzoic acid. After stirred for 10 min, the mixture was heated to 90 °C and further reacted for 5 h. The crude product was collected and washed with DMF by centrifugation at 10,000 rpm for 15 min. Finally, the as-prepared MOFs were stored in ethanol for further usage.

S1.6 Preparation of Red Blood Cell Membranes (RBCMs)

Blood collected from mice was used to prepare the RBC membrane fragments. Briefly, the collected fresh blood was centrifuged at 3,000 rpm for 10 min at 4 °C to remove the plasma. Then the obtained RBCs were further washed twice using cold PBS, and subsequently suspended in 0.25×PBS for hemolysis treatment for 4 h. The released hemoglobin was removed by centrifugation at 12,000 rpm for 10 min. Next, the obtained light pink pellet was washed with cold PBS for three times and stored at -80 °C for further usage. The RBC membrane proteins were quantified by BCA protein assay kit.

S1.7 Preparation of RBCMs-Coated FTP (FTP@RBCM)

The harvested RBCMs were dispersed in PBS and conducted a strong ultrasonic bath for 1 min at a power of 950 W to form uniform RBC vesicles. Next, FTPs were vigorously mixed with as-prepared RBC vesicles at the mass ratio of 5:1 (TCPP in FTPs to total protein in RBCM protein) in 4 mL PBS, and then treated in ultrasonic bath at 4 °C with a moderate power for 10 min. Subsequently, the resulting FTP@RBCM was collected by centrifugation at 13,000 rpm for 15 min.

S1.8 Coomassie Blue Staining

The samples of RBCM, FTP and FTP@RBCM were heated to 100 °C for 10 min in the loading buffer of sodium dodecyl sulfate-polyacrylamide gel electrophoresis (SDS-PAGE). Then, the protein components of different samples were separated by 10% SDS-PAGE. Subsequently, the SDS-PAGE gel was stained with Coomassie blue for 30 min, then washed with the destaining solution (40% ethanol, 10% acetic acid and 50% deionized water) for 3 h and finally imaged by a ChemiDoc^{TM} MP imaging system.

S1.9 Oxygen Generation

O_2 generation was detected using an oxygen indictor $[Ru(dpp)_3]Cl_2$, the photoluminescence of which can be dynamically quenched by O_2 molecules. Thus, the fluorescence intensity of $[Ru(dpp)_3]Cl_2$ was negatively correlated with O_2 concentration in the solution. In detail, FTP (TCPP: 20 µg) solution containing 2 µM of $[Ru(dpp)_3]Cl_2$ was incubated with 10 mM H_2O_2 for

different times (0, 2, 5, 10, and 15 min). Then, the fluorescence spectrum was recorded from 515 to 845 nm under 480 nm wavelength excitation by a Cary Eclipse fluorescence spectrophotometer.

S1.10 ROS Detection

Singlet oxygen sensor green (SOSG) agent as a detection probe was used to monitor reactive oxygen species (ROS) generation. 1 µL of SOSG (5 mM) methanol solution was added into 1 mL FTP solution (TCPP: 20 µg). After irradiation under 670 nm laser (50 mW cm^{-2}) for different times (0, 30, 60, 90, 120, 150, 180, and 240 s), the fluorescence spectrum from 500 to 750 nm was recorded under 480 nm wavelength excitation. The ROS generation efficiency was further analyzed by calculating F_t/F_0 at 530 nm, where F_0 refers to the initial fluorescence intensity, while F_t refers to the fluorescence intensity after irradiation at different times.

To investigate the role of self-compensation of O_2 based on catalase-like reactivity of FTP on ROS generation. 1 mL of FTP (TCPP: 20 µg) solution containing 10 mM H_2O_2 and 5 µM SOSG probe was mixed and co-incubated for 5 min. Then, the mixture was irradiated under 670 nm laser (50 mW cm^{-2}) for 1 or 2 min. Finally, the ROS generation ability was evaluated as the above methods.

S1.11 Detection of GSH Depletion

Briefly, FTP (TCPP: 20 µg) were added into a certain concentration of GSH solution. After incubation for 2, 4 or 6 h, the mixture was centrifugated at 13,000 rpm for 10 min. Then, the supernatant was collected and detected by a Reduced GSH Assay Kit (Solarbio life science, BC1175) according to manufacturer's instruction through a Spectra Max M5 microplate reader.

S1.12 Fe^{2+} Detection

The *o*-phenanthroline can react with Fe^{2+} to form orange complexes, while its complexes with Fe^{3+} are colorless, which can be used to detect the transformation of Fe^{3+} into Fe^{2+}. Simply, FTP (TCPP: 20 µg) was incubated with GSH solution (100 mM) under oscillation for 6 h. Then 50 µL of *o*-phenanthroline solution (1 mg mL^{-1}) was added into the above mixture. The color changes with different treatments could be observed and taken photos finally.

S1.13 OH Generation

FTP (TCPP: 20 µg) was incubated with GSH solution (100 mM) under oscillation for 6 h. Then, the mixture was centrifugated at 13,000 rpm for 10 min, and subsequently the obtained precipitation was added to acetic acid buffer solution (1 mL, 0.1 M, pH 5.0) containing TMB (1 mM) and various concentrations of H_2O_2. After co-incubation for different time intervals, the absorbance at 652 nm was measured using a Spectra Max M5 microplate reader.

Terephthalic acid (TPA) was also used to investigate · OH generation. Briefly, the 5 mM TPA solution containing 5 mM sodium hydroxide (NaOH) was prepared. Then, the precipitation obtained as aforementioned procedures and 10 mM H_2O_2 were added to the mixture (2 mL, pH 6.5). After incubation for 20 min, the fluorescence spectrum from 350 to 600 nm was recorded under 312 nm wavelength excitation by a Cary Eclipse fluorescence spectrophotometer.

S1.14 Cellular Internalization

FTP (TCPP: 400 µg) was dispersed in an aqueous solution containing 20 µM of Cy5. After oscillation for 2 h, the precipitates (Cy5 loaded FTP) were obtained by centrifugation at 13,000 rpm for 10 min and subsequently washed with PBS to remove free Cy5. The Cy5 labelled FTP was further coated with RBCMs or RBCMs pre-treated under 80 °C for 10 min (defined as h-RBCM) to obtain Cy5 labeled FTP@RBCM or FTP@h-RBCM. Subsequently, different cells were seeded at the density of 2 × 10^5 cells and then incubated for 24 h at standard condition. Then the cells were co-cultured with Cy5 labeled FTP@RBCM (TCPP: 80 µg mL^{-1}) for 4 or 8

h. Afterward, the cells washed with PBS, fixed with 4% paraformaldehyde, stained with DAPI and finally observed under CLSM (Zeiss LSM780).

S1.15 Hypoxic Relief

The oxygen indictor of $[Ru(dpp)_3]Cl_2$ was also used to detect intracellular oxygen level. First, the cells were seeded in a 96-well plate at the density of 2 × 10^4 cells per well and cultured for 24 h. Next, the medium was replaced with fresh medium containing FTP@RBCM or FT@RBCM (TCPP: 80 μg mL^{-1}) and further incubated for 8 h. After being washed with PBS for two times, the cells were incubated for another 24 h in normoxia (20% oxygen) or hypoxia (2% oxygen). Then, 2 μM of $[Ru(dpp)_3]Cl_2$ was carefully added into the cells for 30 min incubation. Finally, the cells were washed with PBS for two times and imaged by a fluorescence microscope (Zeiss Axio Vert.A1, Germany).

S1.16 Intracellular ROS Detection

The cells were seeded in a 96-well plate at the density of 2 × 10^4 cells per well and allowed to culture for 24 h in normoxia (20% oxygen) or hypoxia (2% oxygen). Then, the medium was replaced with fresh medium containing FTP@RBCM or FT@RBCM (TCPP: 80 μg mL^{-1}) and further incubated for 8 h in normoxia (20% oxygen) or hypoxia (2% oxygen). After that, the cells were washed with PBS and the medium containing 40 μM DCFH-DA (an intracellular ROS detection probe) were added for another 15 min of incubation. Next, the cells were washed twice with PBS and irradiated under a 670 nm laser (50 mW cm^{-2}) for 3 min. Finally, the cells were observed under a fluorescence microscope.

S1.17 Intracellular GSH Depletion

The cells were seeded in a 6-well plate at a density of 4 × 10^5 cells and cultured for 24 h at standard condition. Then, different concentrations of FTP@RBCM were added into the cells and incubated for 8 h. After wash with PBS for three times, the cells were collected by centrifugation. Subsequently, the concentration of intracellular GSH was detected according to instruction of Reduced GSH Assay Kit.

S1.18 *In Vitro* CCK-8 Assay

To assess the cytotoxicity induced by ·OH produced from the Fenton reaction of FTP@RBCM. The cells were seeded in the 96-well plate at a density of 2 × 10^4 cells per well and incubated for 24 h. Then, the cells were cultured with fresh medium containing different concentrations of FTP@RBCM and further incubated for another 24 or 48 h. After that, the cells were washed twice with PBS and the cell viability was measured by CCK-8 assay.

To evaluate the anti-cancer efficacy of PDT *in vitro*, cancer cells were seeded in the 96-well plate at a density of 2 × 10^4 cells per well and incubated for 24 h in normoxia (20% oxygen) or hypoxia (2% oxygen). Next, different concentrations of FTP@RBCM were added into the cells, which were cultured for another 8 h in normoxia or hypoxia condition. Then, the cells were irradiated under a 670 nm laser (50 mW cm^{-2}) for 5 min. The cells with hypoxic treatment were sealed with 30 μL of paraffin oil to isolate the O_2 in air before irradiation. After another 24 h incubation, the CCK-8 assay kit was used to measure the cell viability.

S1.19 Live/Death Cell Staining Assay

The cells with aforementioned treatment were stained with calcein AM and PI for 30 min and imaged by a fluorescence microscope. It's worth noting that the cell with hypoxic treatment need to be sealed with 30 μL of paraffin oil to isolate the O_2 in air.

S1.20 CRT, HMGB1 Detection and ATP Assay

The cells with aforementioned treatment stained with the primary antibodies of CRT or HMGB1 and corresponding secondary antibodies. Finally, the cells were analyzed by flow cytometry, or stained with DAPI and observed by CLSM (Zeiss LSM780).

The ATP concentration in cells with aforementioned treatment were detected using an ATP assay kit according to the manufacture's instruction.

S1.21 DCs Maturation Assay

DC cells were isolated from bone marrow of mice according to the previous reported literature [S1]. Then, DC cells were cultured with RPMI 1640 medium containing mouse granulocyte/macrophage colony stimulating factor (mGM-CSF, 20 ng mL^{-1}) and IL-4 (10 ng/mL) under standard condition. Afterwards, DC cells were added into the culture medium collected from the aforementioned treatment. The DC cells treated with LPS was taken as positive control. After 48 h of co-incubation, the cells were stained with the antibody of CD11c-APC (eBioscience™, 17-0114-82), CD80-PE (eBioscience™, 12-0801-82) and CD86-PE-Cy7 (eBioscience™, 25-0862-82), then analyzed by flow cytometry.

S1.22 Hemolysis Test

The collected fresh mouse blood was washed twice with PBS, and then centrifugated at 3,000 rpm for 10 min to obtain red blood cells. Subsequently, the red blood cells were incubated with various concentrations of FTP@RBCM at 37 °C for 5 h. The red blood cells incubated with ddH_2O and PBS were used as positive and negative control. After that, the mixture in the tube was centrifugated at 3,000 rpm for 10 min and taken photos. Besides, the supernatants were collected, and then the absorbance at 570 nm of which were measured using a Spectra Max M5 microplate reader. The hemolysis rates were calculated according the below formula:

$$\text{Hemolysis rate (\%)} = (A_1 - A_{negative}) / (A_{positive} - A_{negative}) \times 100\%$$

Herein, A_1 is the absorbance value of sample with different concentrations of FTP@RBCM treatment. $A_{negative}$ and $A_{positive}$ are respectively the absorbance values of negative and positive groups at 570 nm. The final absorbance value of each sample was the averaged value from three independent repetitions.

S1.23 *In Vivo* Bio-Distribution

The C57BL/6 mice were purchased from China Wushi, Inc. (Shanghai, China). All animal experiments were approved by the Animal Ethics Committee of Mengchao Hepatobiliary Hospital of Fujian Medical University and were conducted according to the institutional guidelines. Hepa1-6 subcutaneous tumor model was constructed by subcutaneous injection of Hepa1-6 cells (1×10^6) in the rear right flanks of mice. The mice were fed for animal experiment when the volume of tumor reached about 50 mm^3.

The tumor-bearing mice were intravenously injected with ICG labeled FTP or FTP@RBCM (TCPP: 5 mg kg^{-1}). Then, the mice were imaged using the UniNano- NIR II system at different time points. After 48 h of injection, the mice were sacrificed, and the major organs and tumors were excised to observe the fluorescence distribution of ICG in major organs.

S1.24 *In Vivo* Enhanced Therapeutic Efficacy and Immune Response

he Hepa1-6 tumor-bearing mice were randomly divided into 5 groups including PBS group, PBS + L group, FT@RBCM + L group, FTP@RBCM group and FTP@RBCM + L group. Then at day 0 and 3, the mice were intravenously (*i.v.*) injected with different formulations with the TCPP dose of 5 mg kg^{-1}. After 5 h of enrichment, the mice were conducted with 670 nm laser irradiation (100 mW cm^{-2}) for 10 min. The tumor volumes and mice body weights were measure every other day. The tumor volumes were calculated following the formula:

$$V = \text{length} \times \text{width}^2 / 2$$

At day 20, the tumors and major organs were excised from mice. And the tumors were weighed and recorded, following fixed in formalin for hematoxylin and eosin (H&E), Ki67 and TUNEL staining.

To evaluate the immune response of CRT expression and DC maturation *in vivo*, the tumor-bearing mice were treated with different formulations as above. Next, the tumors and draining inguinal lymph nodes were excised after 3 days of treatment. The tumor tissue sections were used for immunofluorescence staining of CRT. On the other side, the lymph nodes were gently ground to collect cells, and then stained with the antibody of CD11c-APC (eBioscience™, 17-0114-82), CD80-PE (eBioscience™, 12-0801-82) and CD86-PE-Cy7 (eBioscience™, 25-0862-82) for FACS measurement.

S1.25 *In Vivo* Enhanced Synergistic Anti-Tumor Efficacy and Abscopal Effect

To establish the bilateral Hepa1-6 tumor model, the right flanks of mice were subcutaneously injected with 1×10^6 Hepa1-6 cells as the primary tumors, while the left flanks were subcutaneously injected with 5×10^5 cells as the distant tumors. When the volumes of primary tumors reached about 100 mm^3 while the distant tumors were reached about 50 mm^3, the mice were randomly divided into 4 groups including PBS group, FTP@RBCM + L group, anti-Tim-3 group and FTP@RBCM + L + anti-Tim-3 group. The primary tumors of mice were intratumorally injected with 50 μL of FTP@RBCM (TCPP: 2 mg/kg). After 5 h, primary tumors were irradiated with 670 nm laser (100 mW cm^{-2}) for 10 min. On day 1, 4 and 7 after irradiation, the anti-Tim-3 antibodies were *i.p.* injected at the dose of 100 µg per mouse. The tumor volumes in both flanks and body weights of mice were all monitored every other day. Mice were sacrificed when the tumor volume reached or exceeded 1500 mm^3, or when the health condition of mice was impaired seriously.

To analyze the frequency of various lymphocytes in tumors or spleens of mice with different treatments. The bilateral Hepa1-6 tumor-bearing mice were differently treated as described above. The tumors and spleens were excised at 3 days later of the last anti-Tim-3 antibody administration. Then, the primary tumors were digested using collagenase type IV (1 mg/mL), hyaluronidase (0.2 mg mL^{-1}), DNase I (0.02 mg mL^{-1}) at 37 °C for 2 h. The single-cell suspension was obtained after filtered using a cell strainer. a part of the single-cell suspension was stained with anti-CD3-APC (eBioscience™, 17-0032-82), anti-CD4-FITC (eBioscience™, 11-0042-85) and anti-CD8-PE (eBioscience™, 12-0081-82) antibodies for the analysis of T cell infiltration, anti-CD11b-APC (eBioscience™, 17-0112-82) and anti-Ly-6G/C-PE (eBioscience™, 12-5931-82) antibodies for MDSCs analysis. The remaining single-cell suspension was subsequently collected by centrifugation at 800×g for 5 min, and further purified by the density gradient centrifugation using 30% percoll separating solution at 800×g for 30 min. Next, the harvested cells were examined by flow cytometry after staining with anti-CD11b-APC (eBioscience™, 17-0112-82), CD80-PE (eBioscience™, 12-0801-82) or CD206-PE (eBioscience™, 12-2069-42) antibodies for macrophage analysis, CD11c-APC (eBioscience™, 17-0114-82) antibodies for DCs analysis. The distant tumor tissues were fixed and sectioned for immunofluorescence staining of $CD4^+$ and $CD8^+$ T cells. The cells were flushed out from spleens by PBS using a syringe and then passed through 40 µm cell strainer. Next, the cells were collected by centrifugation at 800×g for 5 min and suspended in red blood lysis buffer for 5 min to lyse red blood cells. Afterwards, the cells collected and washed with PBS by centrifugation at 800×g for 5 min, and then purified by the density gradient centrifugation using Ficoll. Subsequently, the harvested cells were stained with anti-CD3-APC (eBioscience™, 17-0032-82), anti-CD4-FITC (eBioscience™, 11-0042-85) and anti-CD8-PE (eBioscience™, 12-0081-82) for T cell population analysis, additional anti-CD44-PE-Cy7 (eBioscience™, 25-0441-82) and anti-CD62L-PerCP-Cy5.5 (eBioscience™, 45-0621-82) antibodies for T memory cell analysis.

S1.26 Cytokine Assay

The tumors were isolated from mice after 3 days of treatment. Then, the tumors weighed about 20 mg were homogenized in 0.5 mL of PBS containing protease inhibitor cocktail. The supernatants were obtained by centrifugation at 10,000 rpm for 15 min. Finally, the cytokines in supernatants were detected using Mouse ELISA Kit (IFN-γ, Boster, EK0375; IL-12, Boster, EK0422; Granzyme B, Boster, EK0417).

S.1.27 Statistical Analysis

Data was presented as the mean ± standard deviation (SD) or min to max, show all points as indicated. Significance was calculated using one-way analysis of variance (ANOVA) or t test as indicated. Survival rates of different groups were compared using the Log-Rank test. Prism 6 software (GraphPad) was used to perform all statistical analyses. $^*P < 0.05$, $^{**}P < 0.01$, $^{***}P < 0.001$. The P-value < 0.05 was considered as statistically significant.

S2 Supplementary Figures

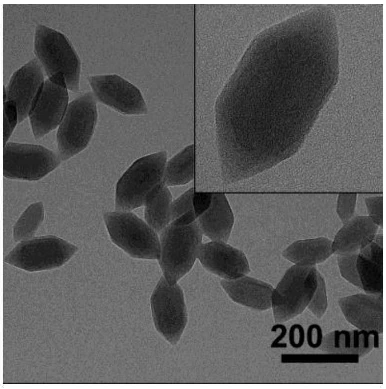

Fig. S1 TEM image of FT

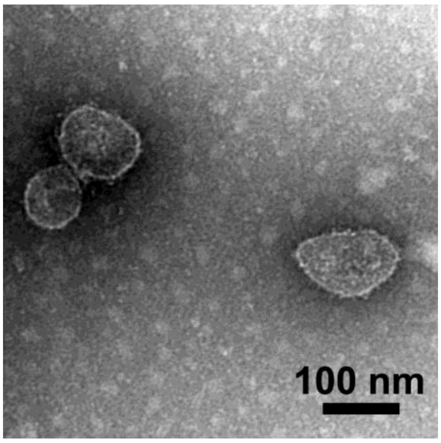

Fig. S2 TEM image of empty RBCM vesicles

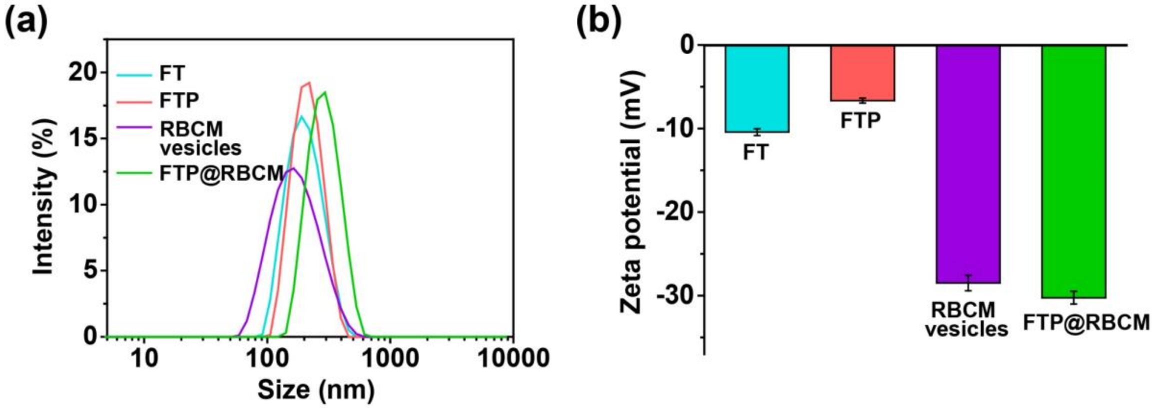

Fig. S3 Size distribution (**a**) and zeta potential (**b**) of different particles

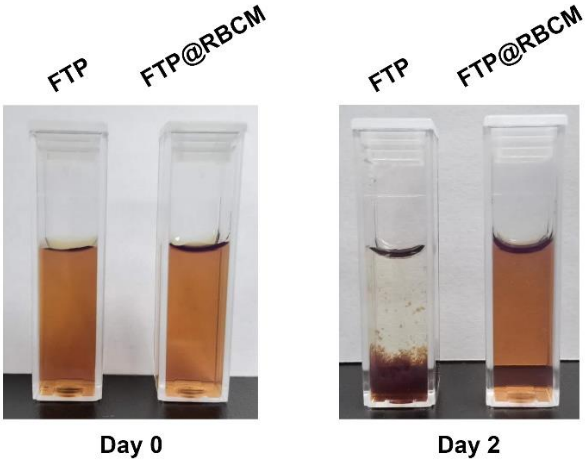

Fig. S4 Photographs of FTP or FTP@RBCM stored in PBS at different time points

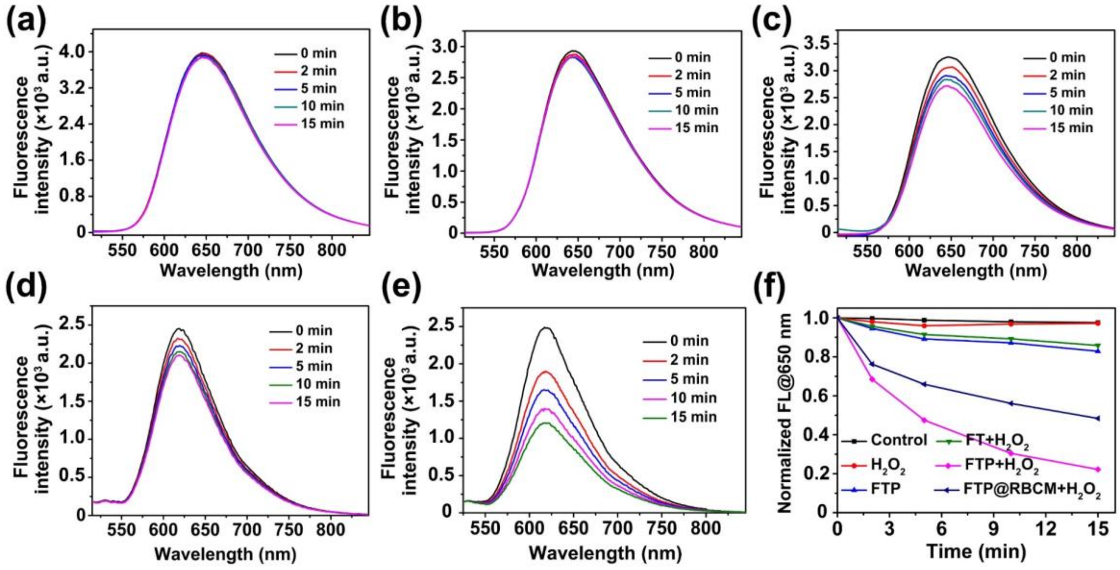

Fig. S5 a The fluorescence spectrum of $[Ru(dpp)_3]Cl_2$ in solution without treatment for different times (Control group). (**b-d**) The fluorescence spectrum of $[Ru(dpp)_3]Cl_2$ in solution after incubation with H_2O_2 (**b**), FTP (**c**), FT + H_2O_2 (**d**) or FTP@RBCM + H_2O_2 (**e**) for different times. (**f**) The normalized fluorescence intensity of $[Ru(dpp)_3]Cl_2$ at 650 nm in different groups

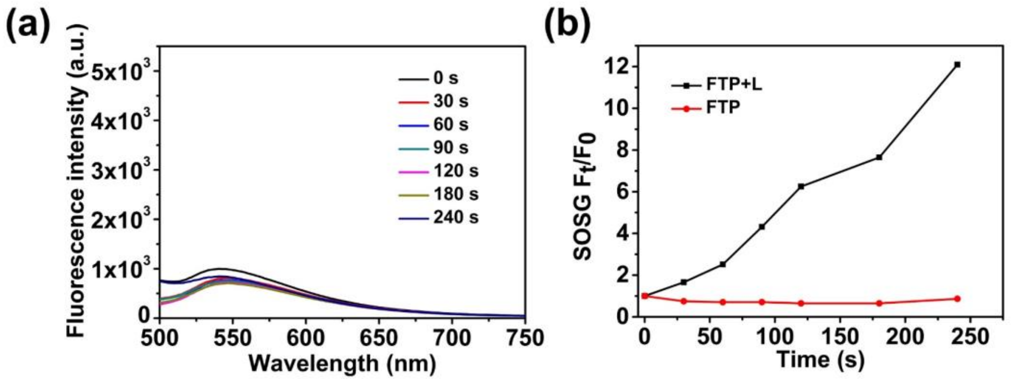

Fig. S6 (**a**) The fluorescence spectra of SOSG in the present of FTP without 670 nm laser irradiation for different times. (**b**) The time-dependence of relative fluorescence intensity of SOSG at 540 nm in different groups

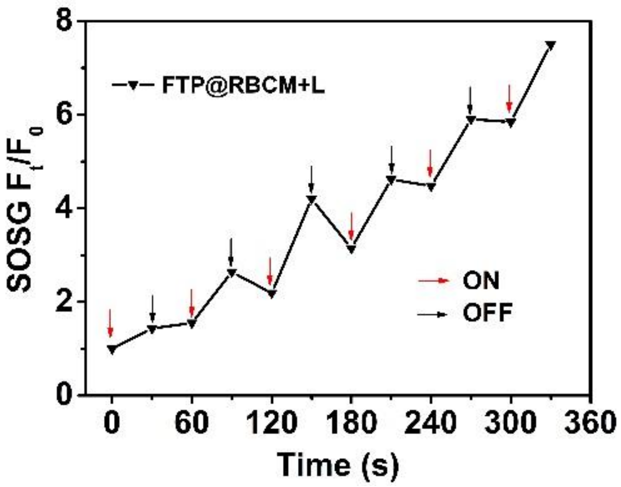

Fig. S7 The relative SOSG fluorescence intensity of FTP@RBCM at 540 nm upon ‘‘ON-to-OFF’’ laser irradiation cycles

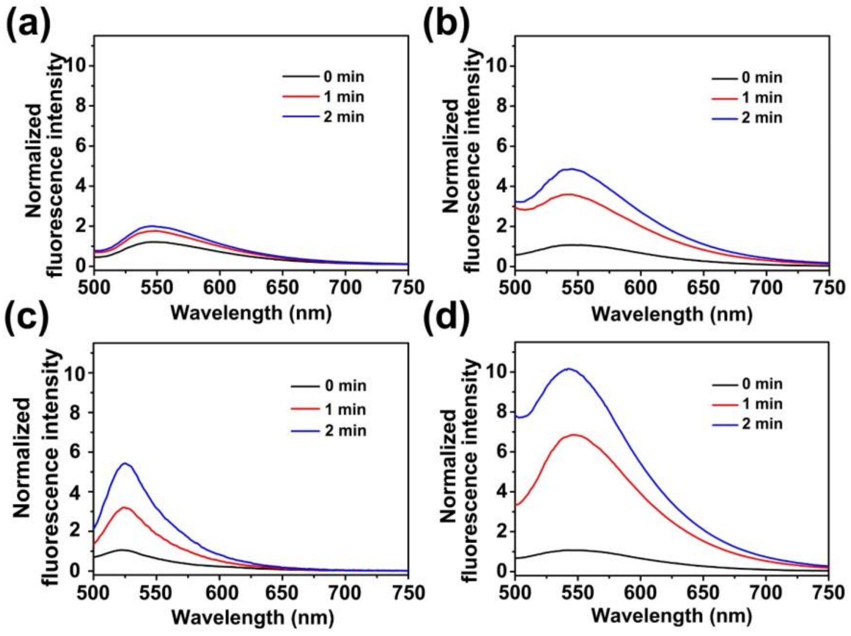

Fig. S8 Fluorescence spectra of SOSG in the solution containing H_2O_2 (**a**), FTP (**b**), FT + H_2O_2 (**c**) or FTP + H_2O_2 (**d**) under 670 nm laser irradiation for 1 or 2 min

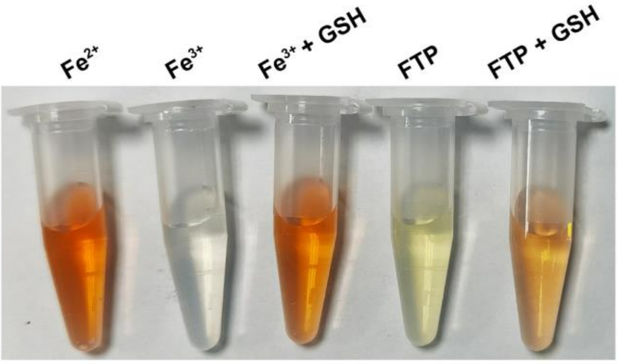

Fig. S9 Photographs of different treatment groups using *o*-phenanthroline as the Fe^{2+} detection probe. The *o*-phenanthroline can react with Fe^{2+} to form orange complexes, while its complexes with Fe^{3+} are colorless

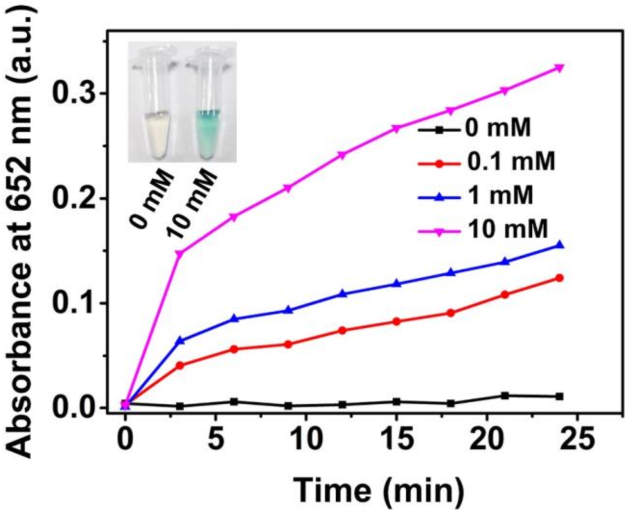

Fig. S10 The absorbance of TMB at 652 nm after incubation with different concentration of H_2O_2 for different times

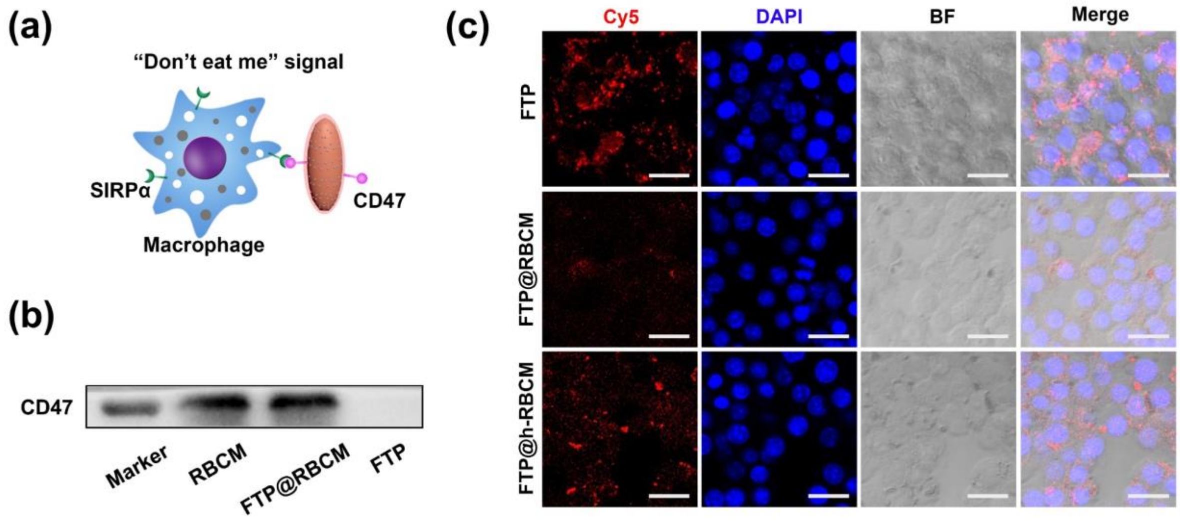

Fig. S11 (**a**) Schematic illustration of macrophage evasion of FTP@RBCM. (**b**) Western blotting analysis of CD47. (**c**) CLSM images of RAW 264.7 cells incubated with Cy5 labeled FTP, FTP@RBCM and FTP@h-RBCM for 4 h. Scale bar = 20 µm

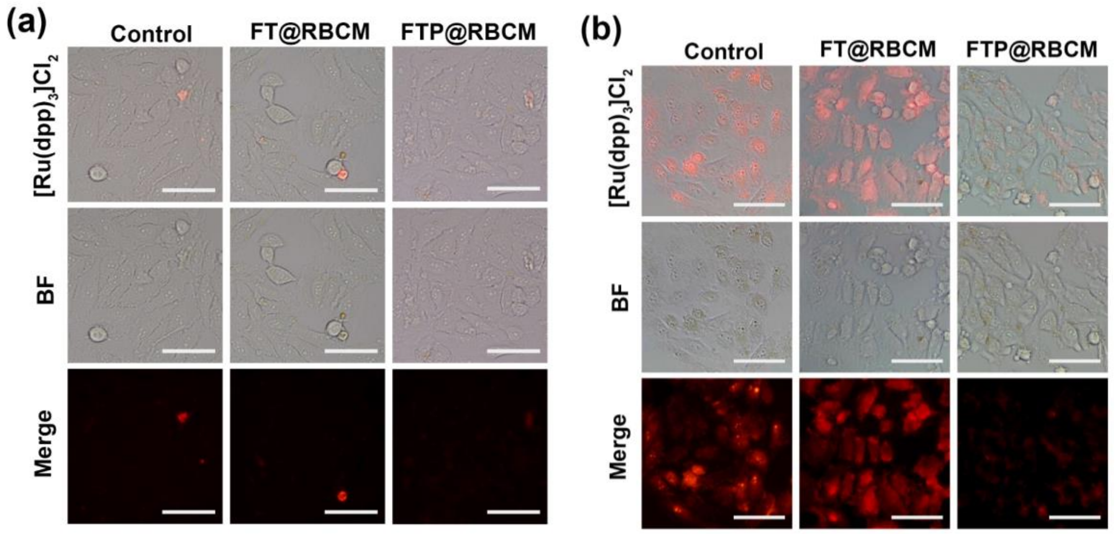

Fig. S12 Fluorescence microscopy images of oxygen detection probe $[Ru(dpp)_3]Cl_2$ in Hep3B cells under normoxia (**a**) and hypoxia (**b**). Scale bar = 50 μm

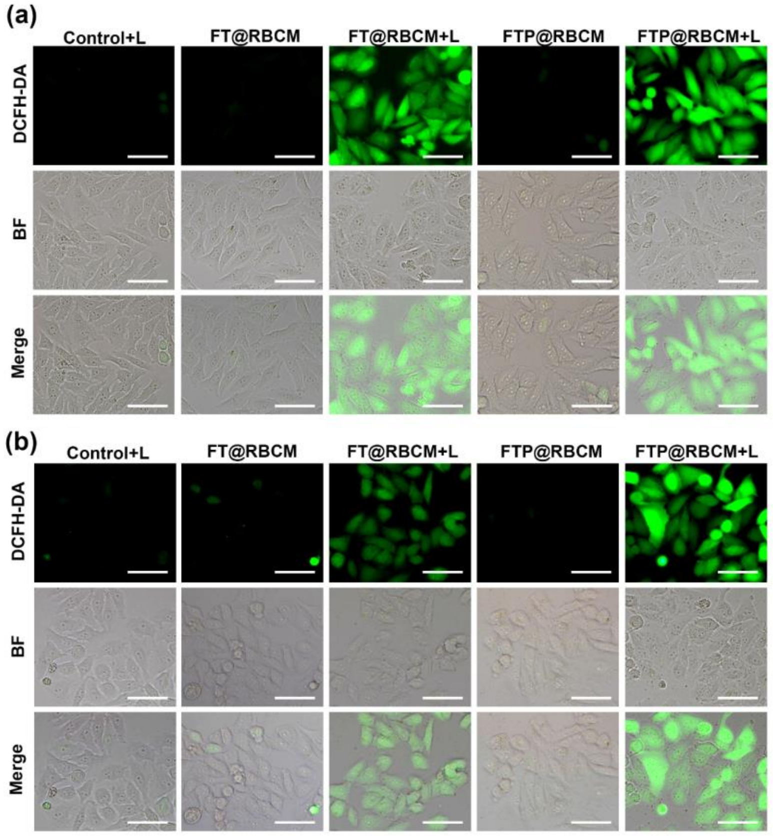

Fig. S13 Fluorescence microscopy images of ROS detection probe DCFH-DA in Hep3B cells under normoxia (**a**) and hypoxia (**b**). Scale bar = 50 μm

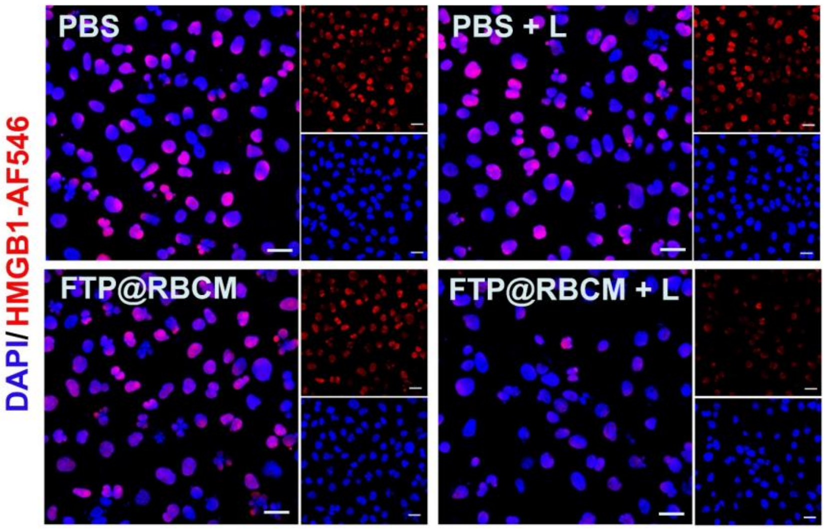

Fig. S14 Uncropped CLSM images in Figure 4b. Scale bar = 20 μm

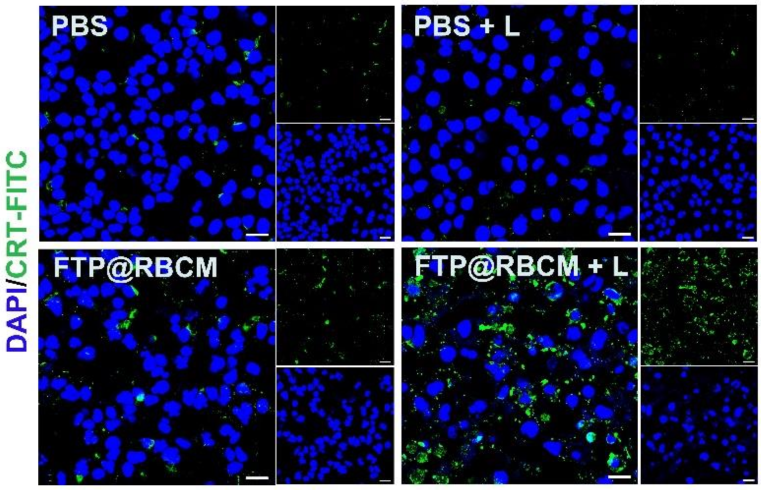

Fig. S15 Uncropped CLSM images in Figure 4d. Scale bar = 20 μm

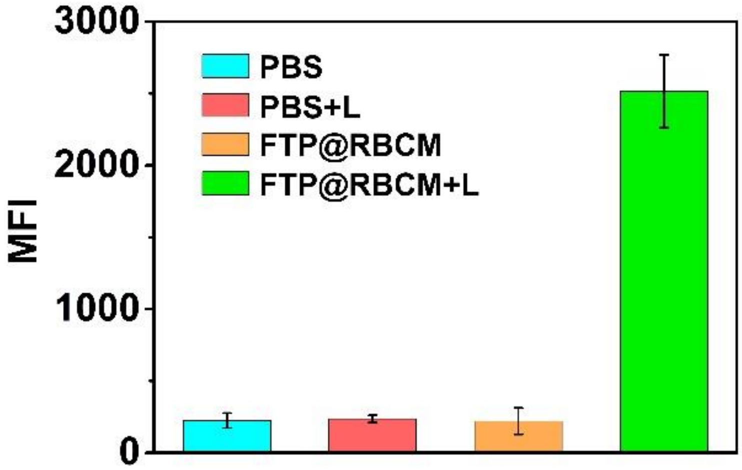

Fig. S16 The corresponding mean fluorescence intensity (MFI) of CRT-FITC in Figure 4e in different groups (n = 3)

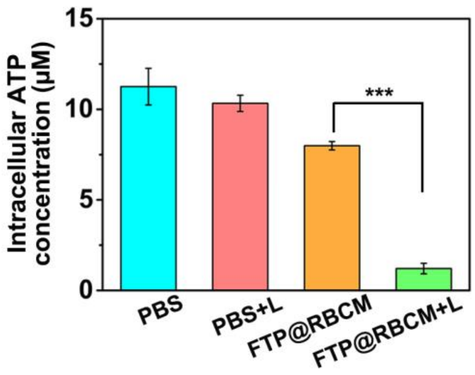

Fig. S17 Intracellular ATP levels of different groups (n = 3)

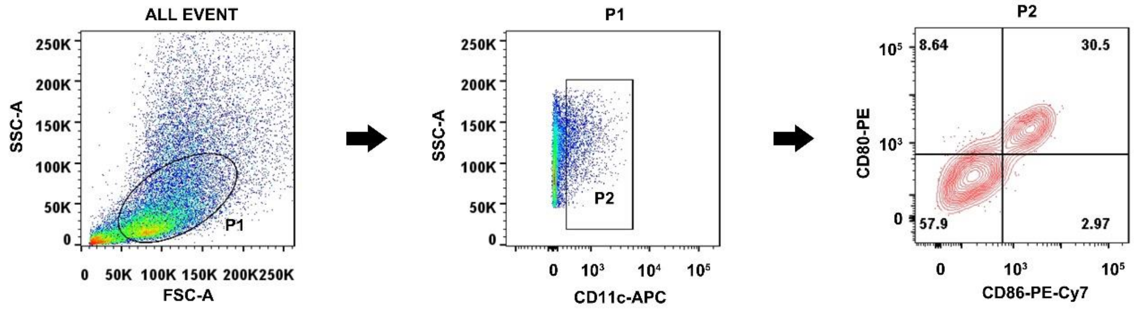

Fig. S18 Representative FACS gating strategy to detect the percentage of matured DCs *in vitro*. Related to Figure 4h

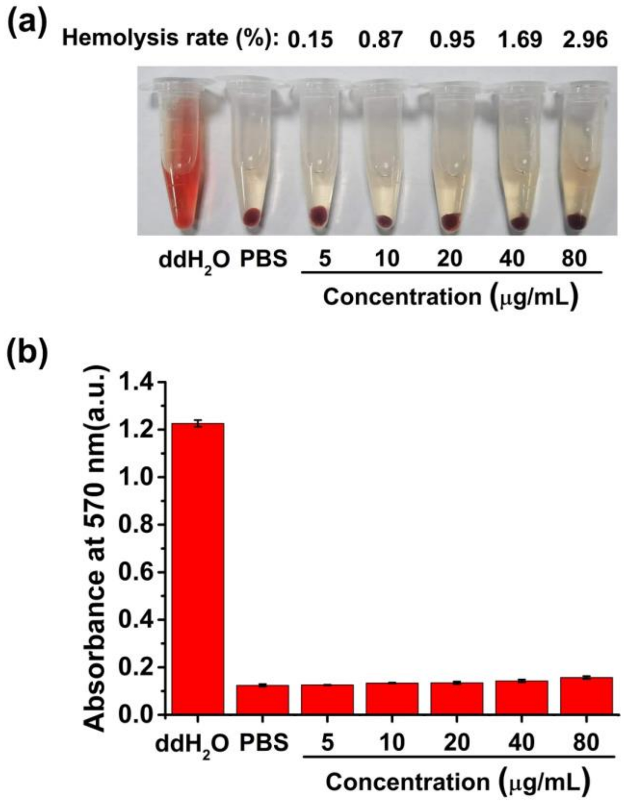

Fig. S19 Hemolysis test of FTP@RBCM with different concentrations. (**a**) The photos of hemolysis test. The tests in PBS and ddH_2O were respectively taken as negative and positive control, respectively. (**b**) The absorbance of supernatants at 570 nm after co-incubation of FTP@RBCM with red blood cells

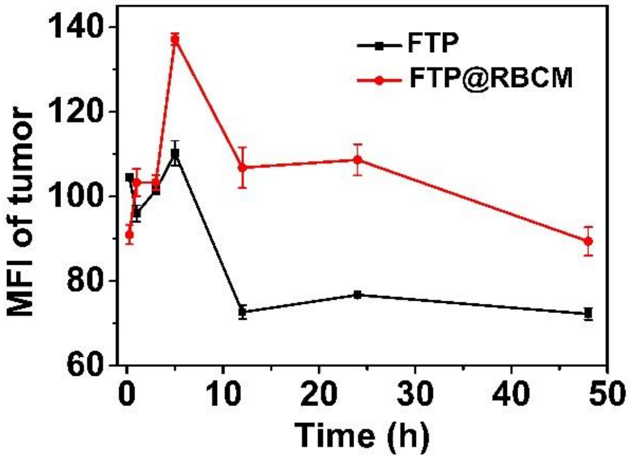

Fig. S20 Quantification of fluorescence intensity of tumor after injection with FTP-ICG or FTP@RBCM-ICG at different time points *in vivo*

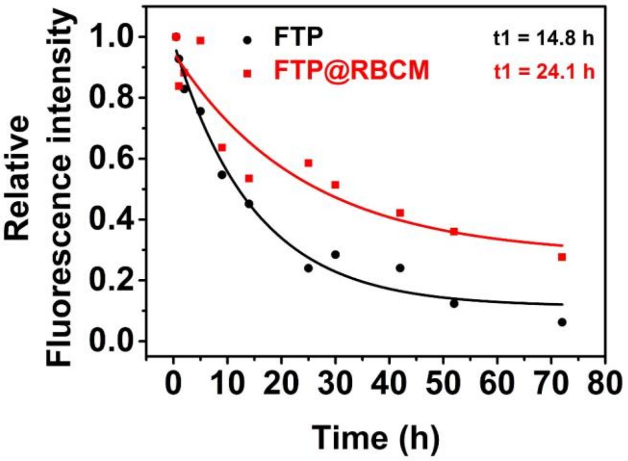

Fig. S21 Relative fluorescence intensity of FTP-ICG and FTP@RBCM-ICG in the serum at different time points after intravenous injection with the half-life time of 14.8 h and 24.1 h, respectively

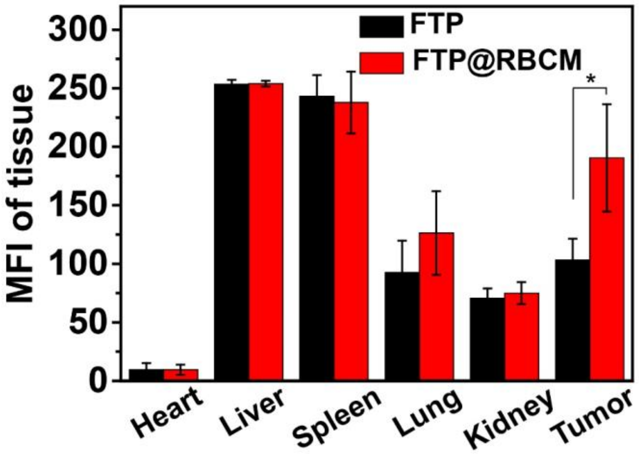

Fig. S22 Quantification of fluorescence intensities of excised main organs and tumors after injection with FTP-ICG or FTP@RBCM-ICG for 48 h

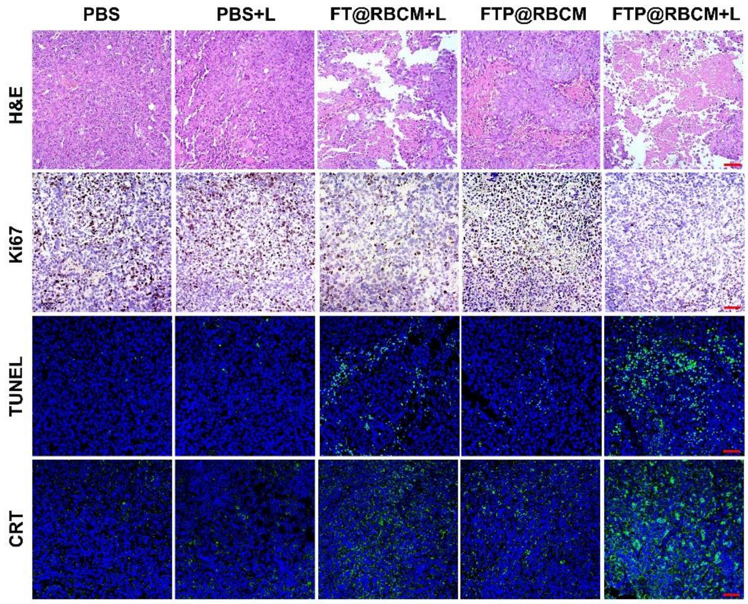

Fig. S23 Uncropped Optical microscope images of H&E and Ki67 antigen immunohistochemistry staining, and TUNEL and CRT immunofluorescence staining in different groups. Related to Fig. 5g. Scale bar = 50 μm

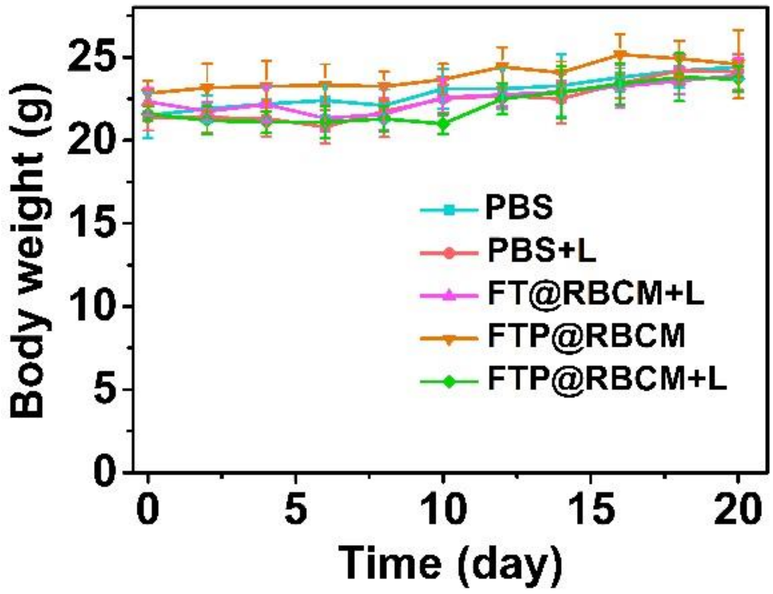

Fig. S24 Average body weights of mice in different groups (n = 5)

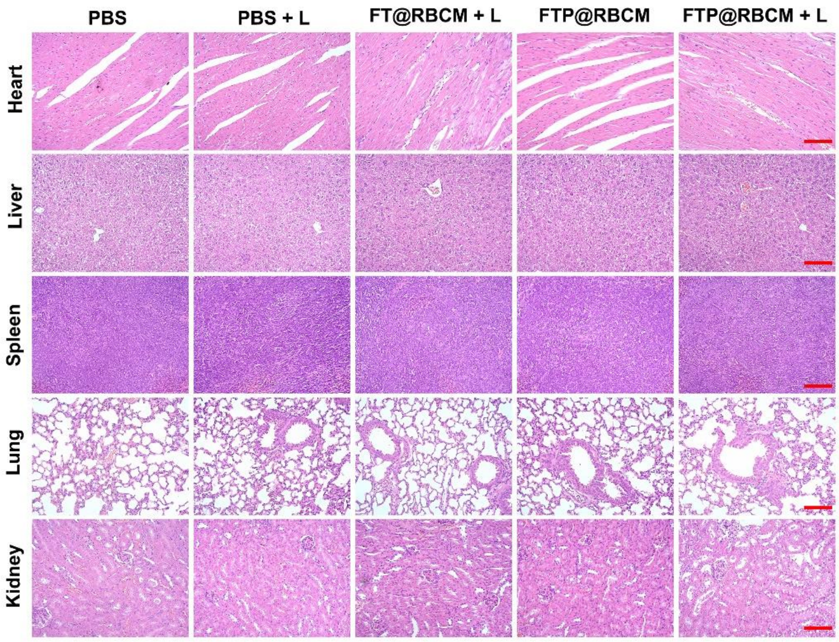

Fig. S25 H&E staining of mice major organs (heart, liver, spleen, lung and kidney) after different treatments. Scale bar = 100 μm

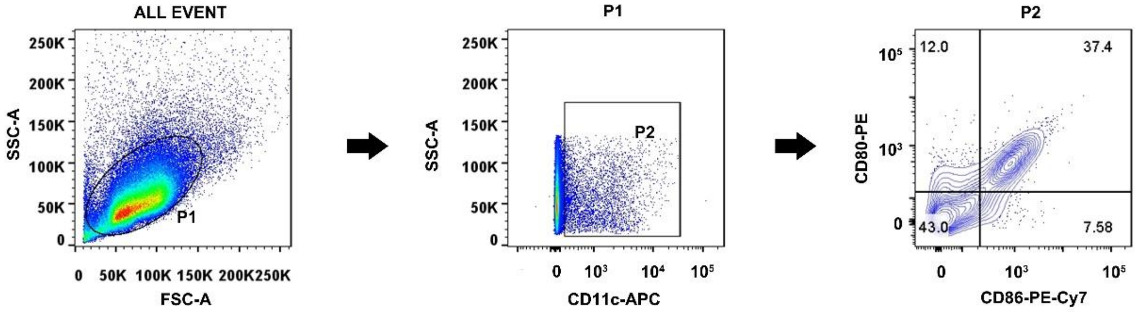

Fig. S26 Representative FACS gating strategy to detect the percentage of matured DCs in draining inguinal lymph nodes of mice. Related to Fig. 5h

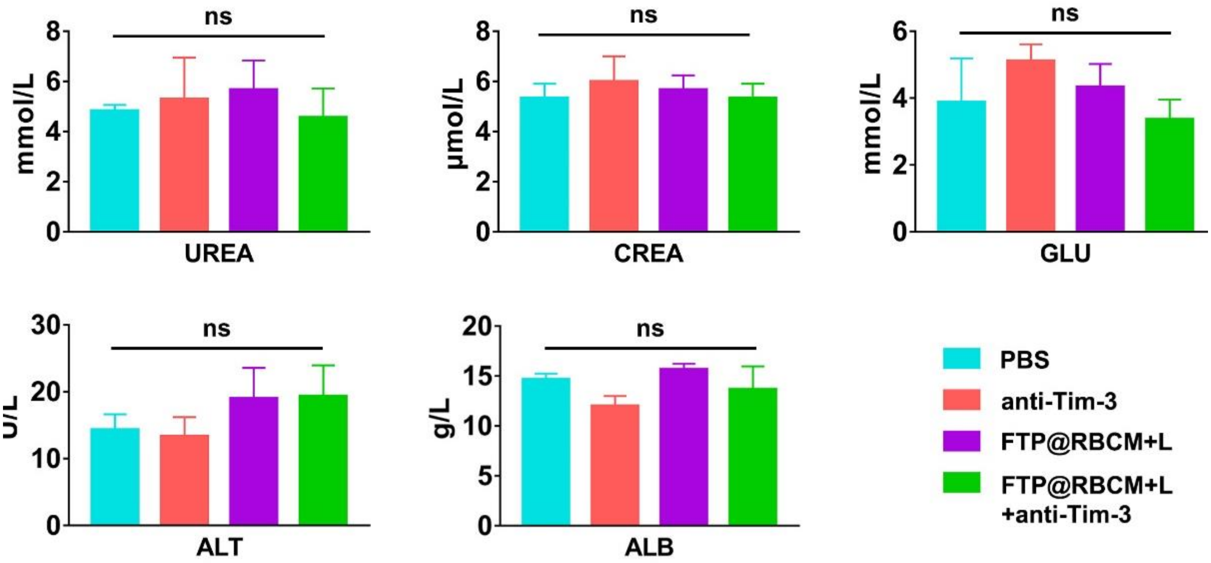

Fig. S27 Serum biochemical indexes (UREA, CREA, GLU, ALT and ALB) of mice after different treatments

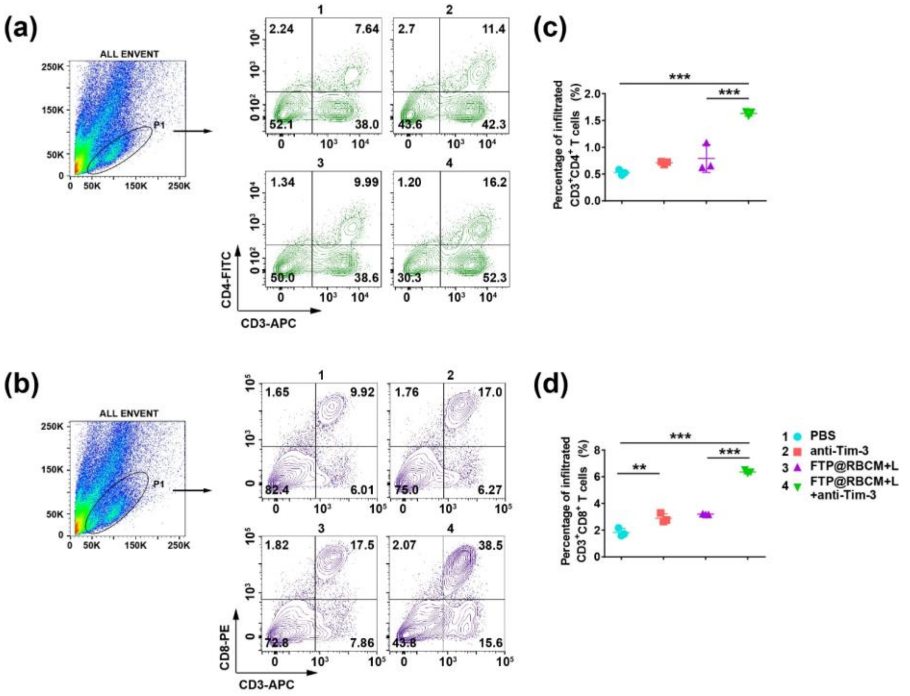

Fig. S28 Flow cytometry analysis (**a, b**) and statistical analysis (**c, d**) of T cells in the primary tumor collected from different groups

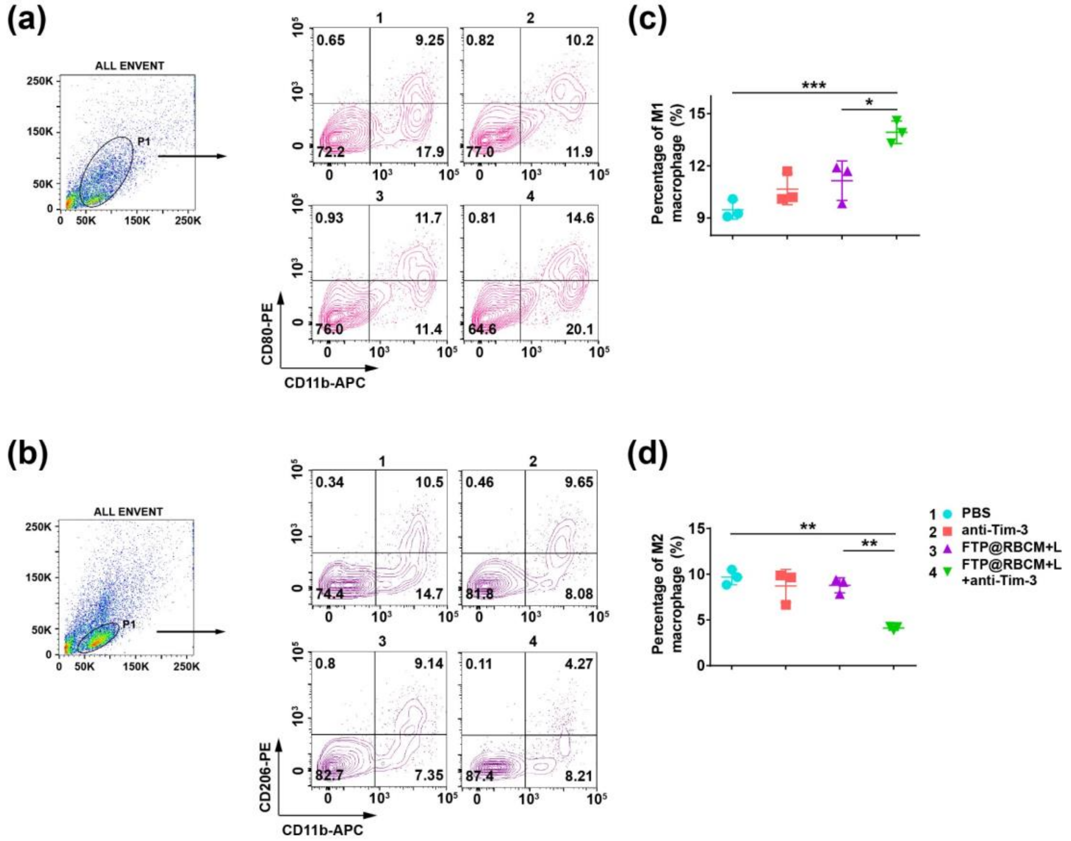

Fig. S29 Flow cytometry analysis (**a, b**) and statistical analysis (**c, d**) of M1 and M2 macrophage in primary tumor collected from different groups

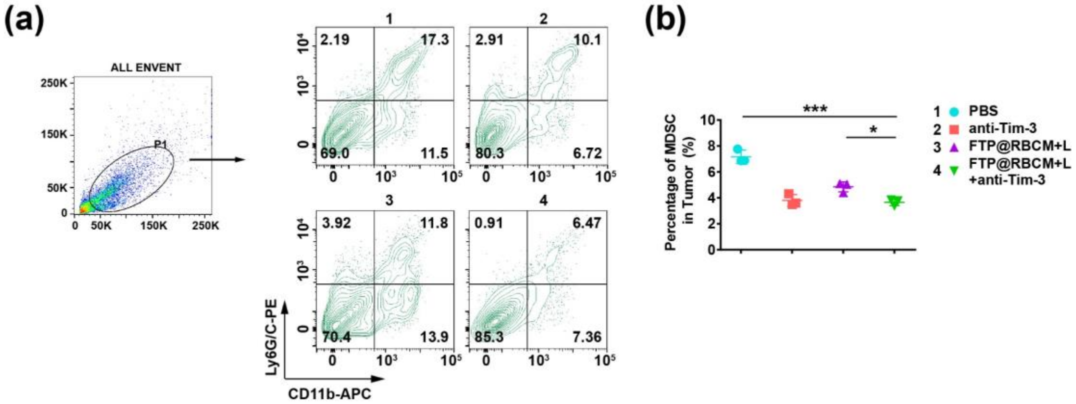

Fig. S30 Flow cytometry analysis (**a**) and statistical analysis (**b**) of MDSCs in primary tumor collected from different groups

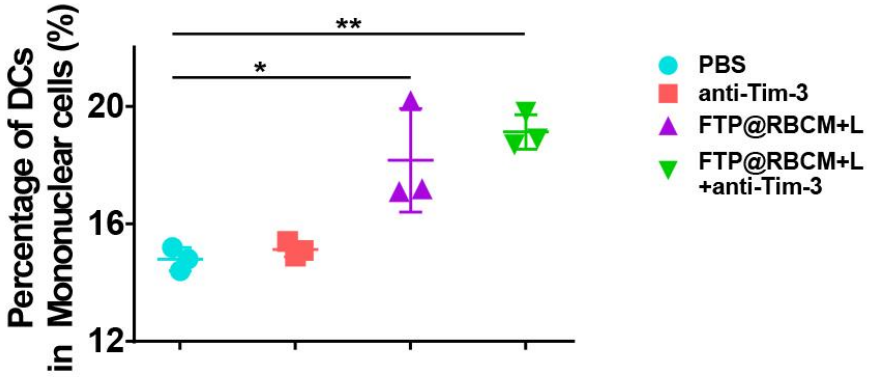

Fig. S31 Statistical analysis of DC cell frequency in mononuclear cells isolated from differently treated mice

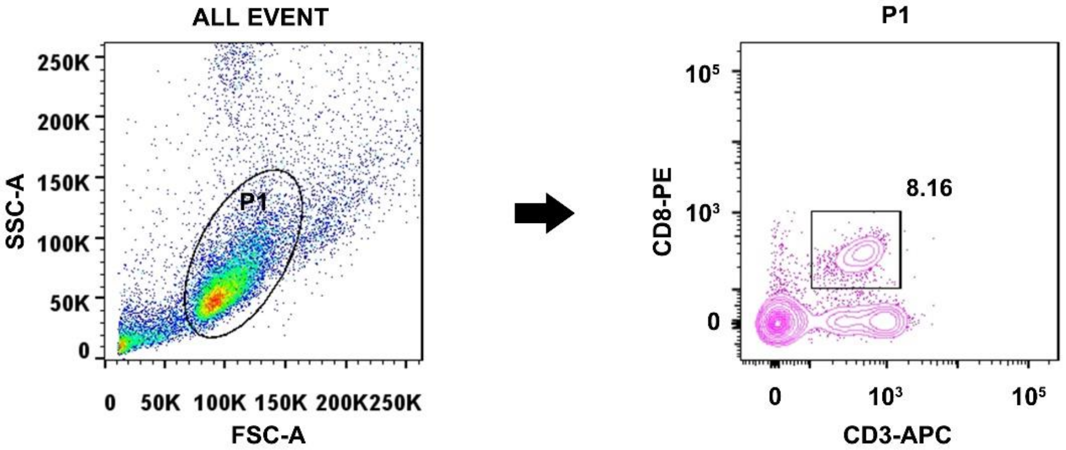

Fig. S32 Representative FACS gating strategy to detect the percentage of $CD3^{+}CD8^{+}$ T cells in spleen. Related to Fig. 6f

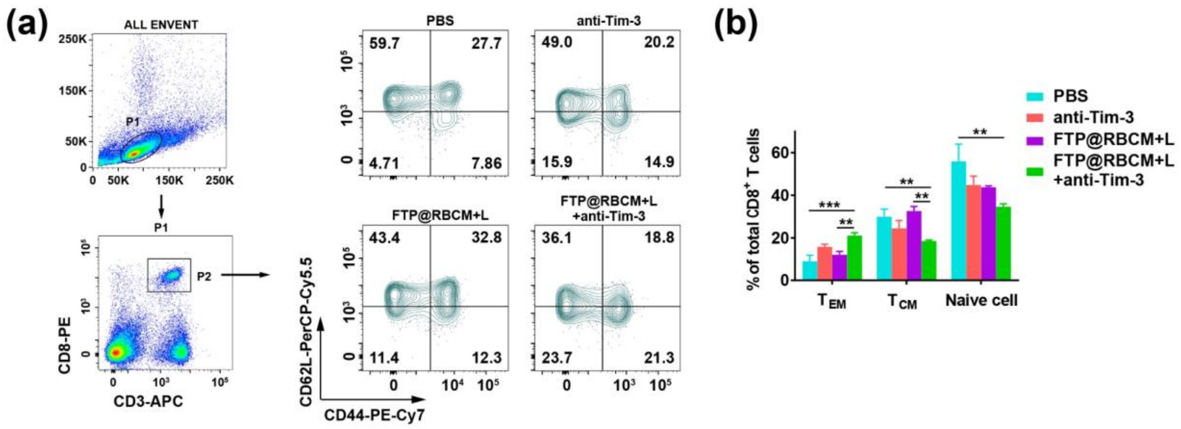

Fig. S33 Flow cytometry analysis (**a**) and statistical analysis (**b**) of the memory CD8$^+$ T cells in spleen isolated from differently treated mice

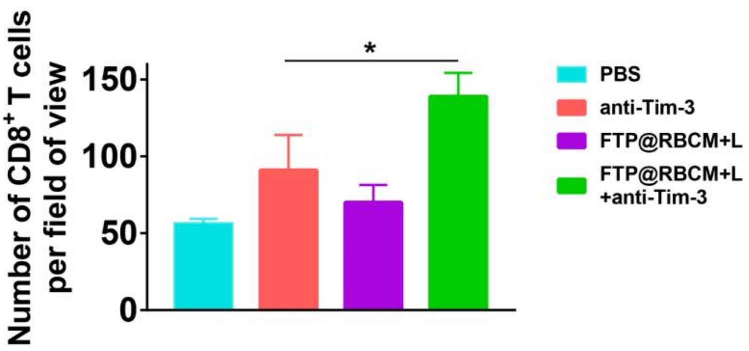

Fig. S34 Quantification of CD8$^+$ T cells from three fields view of each tumor in Fig. 6i

Supplementary References

[S1] M. Wu, D. Zheng, D. Zhang, P. Yu, L. Peng et al., Converting immune cold into hot by biosynthetic functional vesicles to boost systematic antitumor immunity. iScience **23**(7), 101341 (2020). https://doi.org/10.1016/j.isci.2020.101341
